# Adjuvantation of an Influenza Hemagglutinin Antigen with TLR4 and NOD2 Agonists Encapsulated in Poly(D,L-Lactide-Co-Glycolide) Nanoparticles Enhances Immunogenicity and Protection against Lethal Influenza Virus Infection in Mice

**DOI:** 10.3390/vaccines8030519

**Published:** 2020-09-10

**Authors:** Amir Tukhvatulin, Alina Dzharullaeva, Alina Erokhova, Anastasia Zemskaya, Maxim Balyasin, Tatiana Ozharovskaia, Olga Zubkova, Natalia Shevlyagina, Vladimir Zhukhovitsky, Irina Fedyakina, Ivan Pruss, Dmitry Shcheblyakov, Boris Naroditsky, Denis Logunov, Alexander Gintsburg

**Affiliations:** N.F. Gamaleya National Research Center for Epidemiology and Microbiology, 123098 Moscow, Russia; alina-dzharullaeva@yandex.ru (A.D.); erohova.alina95@gmail.com (A.E.); a.zemskaia@mail.ru (A.Z.); b.maxim4432@yandex.ru (M.B.); bu.bulo4ka@gmail.com (T.O.); olga-zubkova@yandex.ru (O.Z.); nataly-123@list.ru (N.S.); zhukhovitsky@rambler.ru (V.Z.); irfed2@mail.ru (I.F.); ivan.pruss@gmail.com (I.P.); sdmitryv@yahoo.com (D.S.); bsnar1941@yahoo.com (B.N.); ldenisy@yahoo.com (D.L.); gintsburg@gamaleya.org (A.G.)

**Keywords:** TLR4, NOD2, PAMPs, complex adjuvant, synergy, pattern-recognition receptors, PLGA, subunit vaccines, nanoparticles, influenza

## Abstract

Along with their excellent safety profiles, subunit vaccines are typically characterized by much weaker immunogenicity and protection efficacy compared to whole-pathogen vaccines. Here, we present an approach aimed at bridging this disadvantage that is based on synergistic collaboration between pattern-recognition receptors (PRRs) belonging to different families. We prepared a model subunit vaccine formulation using an influenza hemagglutinin antigen incorporated into poly-(D,L-lactic-co-glycolic acid) (PLGA) nanoparticles adjuvanted with monophosphoryl lipid A (TLR4 agonist) and muramyl dipeptide (NOD2 agonist). The efficacy studies were conducted in comparison to control vaccine formulations containing individual PRR agonists. We show that the complex adjuvant based on TLR4 and NOD2 agonists potentiates proinflammatory cell responses (measured by activity of transcription factors and cytokine production both in vitro and in vivo) and enhances the phagocytosis of vaccine particles up to comparable levels of influenza virus uptake. Finally, mice immunized with vaccine nanoparticles containing both PRR agonists exhibited enhanced humoral (IgG, hemagglutination-inhibition antibody titers) and cellular (percentage of proliferating CD4+ T-cells, production of IFNɣ) immunity, leading to increased resistance to lethal influenza challenge. These results support the idea that complex adjuvants stimulating different PRRs may present a better alternative to individual PAMP-based adjuvants and could further narrow the gap between the efficacy of subunit versus whole-pathogen vaccines.

## 1. Introduction

Influenza virus remains one of the main threats to humanity, in spite of a long battle to eliminate infection. According to a WHO report, annual epidemics alone are estimated to result in up to 5 million cases of severe illness, and up to 650,000 respiratory deaths [1]. Vaccination is considered to be the most effective strategy to preventing infectious diseases [2]. Choosing the highest safety characteristics as a primary requirement for modern vaccines, it is rational to use single highly purified antigenic molecules for subunit vaccine preparation. Indeed, subunit vaccines containing precisely defined antigenic molecules generally cause fewer side effects than vaccines based on either inactivated or live attenuated whole pathogens. At the same time, a significant reduction in pathogen-specific molecules presents an immune portrait in which subunit vaccines are scarce and, therefore, rather distant from the original pathogen, which, in turn, is reflected by their decreased immunogenicity. Considering this, some “danger” signals associated with pathogens should be brought back into vaccine composition in an attempt to restore immunogenicity without affecting safety characteristics.

Progress in understanding the molecular mechanisms by which the innate immune response is induced has led to the identification of such patterns of “danger” in pathogen structures called pathogen-associated molecular patterns (PAMPs) [3]. The binding of PAMPs by pattern-recognition receptors (PRRs) initiates intracellular signaling cascades, eventually inducing the expression of a variety of proinflammatory molecules, which assemble together to participate in the innate and adaptive immune response aimed at eradication of the infection. This fundamental process is the basis of a long-awaited rational approach for creating and selecting new types of adjuvants based on highly purified and characterized PAMP-containing molecules. Appearing to be generally safe and well-tolerated, PAMP-containing adjuvants significantly enhance antigen immunogenicity, which makes them indispensable in modern subunit vaccines. Today, many adjuvants stimulating specified PRRs are completing preclinical and clinical studies, where four adjuvants (AS01, AS02, AS04, and MF59) based on the TLR4 agonist monophosphoryl lipid A (MPLA) have already been registered for clinical use [4].

Meanwhile, immune reactions followed by the activation of individual PRRs seem to be scarcely feasible events in the conditions of real infection. Acting as a complex of various PAMPs, pathogens likely activate sets of different PRRs during their invasion process [5]. Specifically, the influenza virus could be recognized by members of at least three pattern-recognition receptor families, including TLR7, RIG-I, and NLRP3 [6]. This implies that the immune response elicited during infection (aimed toward the most efficient pathogen elimination) could be distinct from more ‘artificial’ ones induced by the triggering individual PRR types. It has been shown that the combined stimulation of PRRs belonging to distinct families, for example, transmembrane Toll- and cytosolic nucleotide-binding oligomerization domain (NOD)-like receptors not only enhances the magnitude of cell response but also defines unique properties not observed after individual PRR stimulation, such as (i) an ability to maintain protective immune response via unchanged NOD1/2 signaling under LPS-tolerant conditions (and vice versa) [7], (ii) a broadening profile of cell types involved in immune response with the limited PRR repertoire [8,9], and (iii) an ability to secrete IL-1β and IL-18 cytokines that require a two-step mechanism of production [10].

Such collaboration phenomenon between TLR and NOD-like receptors provides possibilities for significant improvement in subunit vaccine immunogenicity by using complex adjuvants containing combinations of PAMPs. In our previous study, we showed that the combination of TLR4 (MPLA) and NOD2 (muramyldipeptide (MDP)) agonists adsorbed on alum particles together with ovalbumin (taken as a model antigen) significantly improves the phagocytosis of model vaccine particles and the maturation of dendritic cells (DCs), and enhances the activation of both the humoral and cell-mediated adaptive immune response [11]. However, whether such an approach using complex immunoadjuvants based on the combination of TLR and NOD receptor agonists could result in vaccine protective efficacy still remains unstudied.

For this purpose, we chose recombinant hemagglutinin (HA) from pandemic influenza virus A/California/07/2009 (H1N1) as a protective antigen adjuvanted with MPLA and MDP individually or in combination. To co-localize the antigen and adjuvant molecules we chose poly-lactic-co-glycolic acid (PLGA) polymer as a particulate carrier according to several advantages favorably distinguishing it among many others: (i) High safety characteristics and biocompatibility, (ii) versatile technology allows to efficiently encapsulate into PLGA particles molecules with widely differing physicochemical properties, (iii) prepared highly stable solid PLGA nanoparticles could be easily quantified and standardized (e.g., by size and content), and (iv) being biologically inert PLGA allows to study effects of vaccine adjuvantation without impact of vaccine carrier (e.g., compared to widely used Alum in subunit vaccine formulations that activates the NLRP3 inflammasome pathway [12]). 

Proceeding from this we show here for the first time that vaccine particles containing complex PAMP-based adjuvant showed higher immune response compared to vaccine particles adjuvanted with individual PRR agonists used separately or even equally mixed. Such combined stimulation of TLR4 and NOD2 during immunization makes a significant impact on protection against lethal influenza infection compared to individual PRR stimulation. We also estimated efficiency of the engulfment of PAMP-adjuvanted PLGA NPs (equally sized and previously normalized by number) by dendritic cells in comparison to live H1N1 influenza particles taken as an example of live attenuated influenza vaccine (LAIV). Obtained results demonstrate that adjuvantation with both PRR agonists maximize the uptake levels of subunit vaccine particles reaching the levels comparable to LAIV particles. These data bring the opportunity to design subunit vaccines equally immunogenic to classical whole-pathogen-based analogs meeting with high standards of safety requirements as well.

## 2. Materials and Methods

### 2.1. Cell Lines

RAW-Blue, HEK-Blue-hTLR4, HEK-Blue-hNOD2, and control HEK-Blue-Null2 cells were obtained from Invivogen (San Diego, CA, USA) and maintained in Dulbecco‘s Modified Eagle’s medium (DMEM) medium (GE Healthcare, Chicago, IL, USA) supplemented with 10% fetal calf serum (Thermo Scientific, Waltham, MA, USA), 50 U/mL penicillin, 50 μg/mL streptomycin, 2 mM glutamine, and 0.1 M NaHCO_3_ (all PanEco, Moscow, Russia) at 37 °C with 5% CO_2_.

### 2.2. Preparation of PLGA NPs

Copolymer poly(D,L-lactide-co-glycolide) (PLGA) NPs were produced by the double emulsification solvent evaporation technique, as previously reported [13]. Briefly, 100 mg of 75:25 PLGA (Lactel, Birmingham, AL, USA) was dissolved in 5 mL ethyl acetate (Sigma-Aldrich, Saint Louis, MO, USA), as a non-polar solvent, in a 50 mL centrifuge tube (Corning, New York, NY, USA). Tubes were placed into an ultrasonic bath for 30 min until all of the polymer was completely dissolved. An aqueous phase comprising 40 μg hemagglutinin (HA) protein of the influenza virus HA(∆TM)(A/California/07/2009(H1N1)) (Immune Technology Corp, New York, NY, USA) was dissolved in 500 μL of 1× phosphate-buffered saline (Sigma-Aldrich, Saint Louis, MO, USA) alone or with the immunostimulatory molecules MPLA (350 μg) (Sigma-Aldrich, Saint Louis, MO, USA) and MDP (1.25 mg) (Invitrogen, San Diego, CA, USA) either individually or in combination. Another aqueous phase was added to the tube containing PLGA in a dropwise manner and sonicated on ice using a Branson 450 Digital Sonifier (Emerson Electric Co, Sait Louis, MO, USA) at 70 W for 30 s. A second polar phase represented by 20 mL of 1% *w/v* polyvinyl alcohol (PVA) solution in mQ water was added to the resulting primary emulsion. Samples were placed in ice and sonicated at 70 W for 30 s. The prepared double emulsion was stirred on a magnetic stirrer overnight at 25 °C to evaporate the ethyl acetate. After centrifugation (2000 *g*, 30 min, 4 °C), the supernatant containing PLGA NPs was collected. PLGA nanoparticles were sedimented (10,000 *g*, 30 min, 4 °C) and washed five times with mQ water to remove the residual PVA. PLGA suspension was aliquoted (5 mg in 2 mL) and 0.1% *w/v* sucrose was added as a cryoprotectant prior to freeze-drying using Freezone Plus, 2.5 L (Labconco, Kansas City, MO, USA). Samples of PLGA NPs were sealed under sterile conditions and stored at −20 °C before use.

### 2.3. Characterization of Physical Parameters of PLGA and Influenza Virus Particles

Average particle size, polydispersity Index (PDI), and zeta-potential measurements were conducted using a Malvern Zetasizer Nano instrument (Malvern Instruments, Malvern, UK) and analyzed with Zetasizer 7.01 software (Malvern Instruments, Malvern, UK). The average particle size and PDI of PLGA NPs diluted to 100 μg/mL in 10 mM Tris-buffer pH 7.4 were determined by the dynamic light scattering (DLS) method in a UV microcuvette (BrandTech, Essex, CT, USA). The zeta-potential (laser Doppler electrophoresis) of PLGA NPs diluted to 1 μg/mL in 10 mM Tris-buffer, pH 7.4, was analyzed in folded capillary cells (Malvern Instruments, Malvern, UK). Virus and PLGA particle quantity as well as size distribution were estimated by nanoparticle tracking analysis (NTA) using a NanoSight NS300 system (Malvern Technologies, Malvern, UK) configured with a 488 nm laser and CMOS camera. PLGA NPs were diluted to 0.1 μg/mL concentration in particle-free 0.02 μm filtered PBS (Whatman, Maidstone, UK). Samples were analyzed under constant flow conditions (flow rate = 60 μL/min) at 25 °C. Data were analyzed using NTA 3.2 software. In order to achieve consistent calculation of particle distribution and concentration, 3 × 60 s videos were captured with a camera level from 9 to 14 and a detection threshold of 4 for PLGA NPs and of 3 for H1N1.

### 2.4. PVA Content

Residual PVA content in PLGA preparations was determined using an iodine–PVA spectrophotometric method as previously reported [14]. Briefly, PLGA samples (1 mg) were dissolved in 0.1 M NaOH as well as standard PVA samples ranging from 31 μg/mL up to 1 mg/mL, and were added into a 96-well plate in triplicates. Afterwards, 140 μL of mQ water, 75 μL of 4% boric acid, and 15 μL of 1.27% iodine with 2.5% potassium iodine was added to each well. The absorbance of all the samples was measured at 670 nm using Synergy H4 (Biotek, Winooski, VT, USA).

### 2.5. Measurement of HA Encapsulated in PLGA Particles

Firstly, PLGA NPs (6 mg per sample) were lysed after resuspension in 1 mL of bio0.1 M NaOH and subsequent sonication in ultrasonic bath (Biosan, Rigas, Latvia) for 30 min. Neutral pH was restored by addition of 0.2M HCl prior measuring the encapsulation efficacy of HA in samples using the Bicinchoninic Acid Protein Assay Kit (AppliChem, Maryland Heights, MO, USA). According to the manufacturer’s instructions, 150 μL of samples were added to 96-well plates containing 75 μL Reagent A, 72 μL Reagent B, and 3 μL Reagent C. For an evaluation of the protein concentration, a calibration curve was prepared using 150 μL samples of HA in concentrations ranging from 1 to 250 μg/mL. A plate was incubated at 37 °C for 30 min. The absorbance of all the samples was measured at 562 nm using Biotek Synergy H4 reader.

### 2.6. Measurement of PRR Agonists Internalized in PLGA Particles

PLGA NPs were lysed and neutralized (as described above) prior to addition to HEK-Blue-hTLR4 and HEK-Blue-hNOD2 reporter cells containing an NF-κB/Ap-1-dependent secreted embryonic alkaline phosphatase (SEAP) reporter construct and artificially expressing hTLR4 and hNOD2 (all Invivogen, San Diego, CA, USA). Soluble MPLA and MDP molecules were used as standards to construct a calibration curve (Figure S1). Parental HEK-Blue-Null2 reporter cells were used as a negative control. SEAP activity was determined in the culture medium as described further.

### 2.7. SEAP Reporter Assay

Reporter cells were seeded in 96-well plates at 5 × 10^4^ cells per well for RAW-Blue cells and 2 × 10^4^ cells per well for HEK-Blue cells (HEK-Blue-hTLR4, HEK-Blue-hNOD2, and HEK-Blue-Null2) in a complete DMEM medium. The next day, cells were treated with intact or lysed and neutralized samples of PLGA NPs or soluble PRR agonists at the indicated doses. Secreted embryonic alkaline phosphatase (SEAP) activity was assayed after 18 h of incubation by mixing 50 μL clarified culture supernatants with 150 μL of 60 μM *p*-nitrophenylphosphate (Sigma-Aldrich, Saint Louis, MO, USA) dissolved in an SEAP assay buffer (0.5 M carbonate, pH 9.8, 0.5 mM MgCl_2_). The absorbance was measured at 405 nm read in a Wallac 1420 plate reader (PerkinElmer, Waltham, MA, USA).

### 2.8. Dendritic Cell Cultures

Bone marrow-derived dendritic cells (BMDCs) from C57BL/6 mice were differentiated from proliferating mouse bone marrow progenitors through induction with 20 ng/mL granulocyte macrophage colony stimulating factor (GM-CSF) (R&D Systems, Minneapolis, MN, USA) over 6–9 days as described [15], with slight changes mentioned in [11]. Prior to further experiments, a percentage of CD11c + MHCII + cells in harvested BMDCs was evaluated by flow cytometry (≈ 60–70%).

### 2.9. In Vivo and Ex Vivo Cytokine Analysis

For cytokine analysis, BMDCs, RAW-Blue cells, and murine splenocytes were seeded in 96-well plates at 2 × 10^5^ cells per well. PLGA or virus particles were added to the plates on the next day (in triplicates). At 24 h after treatment, cell-free culture supernatant samples were obtained for further cytokine analysis. Tissue samples (inguinal lymph nodes, liver, spleen, and thigh muscle) were isolated for ex vivo cytokine assays 3 h after PLGA NP injection. Tissue homogenates were prepared and normalized using a method described in [16]. Concentrations of IL-1α, IL-1β, IL-2, IL-3, IL-4, IL-5, IL-6, IL-9, IL-10, IL-12 (p40), IL-12 (p70), IL-13, IL-17A, eotaxin (CCL11), G-CSF, GM-CSF, IFN-γ, KC (CXCL1), MCP-1 (CCL2), MIP-1α (CCL3), MIP-1β (CCL4), RANTES (CCL5), and TNF-α in the obtained samples were estimated using the mouse 23-plex bead-based Bio-Plex Pro Kit (Bio-Rad Laboratories, Hercules, CA, USA) according to the manufacturer’s instructions.

### 2.10. Analysis of Ifnβ Expression by RT-qPCR

BMDCs were seeded in 12-well plates at 2 × 106 cells per well. PLGA NPs were added to the plates on the next day (in triplicates). At 72 h after treatment, cells were collected and washed with ice-cold PBS. Total RNA was isolated using TRIzol Reagent (Thermo Fisher Scientific, Waltham, MA, USA) following the manufacturer’s protocol. Prior to cDNA preparation, genomic DNA was removed by RQ1 RNase-Free DNase (Promega, Madison, WI, USA). Between 1.5 and 2 μg of total RNA was converted into cDNA using 50 μM of random hexamers primers and SuperScript IV Reverse Transcriptase (Thermo Fisher Scientific, Waltham, MA, USA) according to the product protocol. cDNA equivalent of 150-200ng RNA was used for the amplification of Glyceraldehyde-3-phosphate dehydrogenase (*gapdh*) taken as a reference gene, as well as beta interferon (*ifnβ*) gene with the following primer pairs: *gapdh* gene forward primer, 5’- AACTTTGGCATTGTGGAAGG-3’; *gapdh* gene reverse primer, 5’-ACACATTGGGGGTAGGAACA-3’; *ifnβ* gene forward primer, 5’-TGAATGGAAAGATCAACCTCACCTA-3’; *ifnβ* gene reverse primer, 5’-CTCTTCTGCATCTTCTCCGTCA-3’. RT-qPCR was carried out using qPCRmix-HS SYBR mixture (Evrogen JSC, Moscow, Russia) and analyzed on CFX 96 machine (Bio-Rad Laboratories, Hercules, CA, USA). Control samples without transcriptase or RNA were used to check DNA contamination.

The amplification protocol consisted of 5 min 95 °C followed by 40 cycles of 20 s 95 °C, 20 s 60 °C, and 30 s 72 °C; and completed with a standard melting curve protocol. The data were processed using Bio-Rad SFX software. Relative changes in gene expression levels were determined using the 2^−∆∆Ct^ method.

### 2.11. Influenza and HA pHrodo Labeling

To assess the phagocytosis of PLGA particles, HA was labeled using a pHrodo Red Microscale Labeling Kit (Life Technologies, Carlsbad, CA, USA) according to the manufacturer’s instructions. The labeling efficiency was calculated using a Synergy H4 reader (Biotek, Winooski, VT, USA) according to the manufacturer’s instructions. The labeled HA was then used for the preparation of PLGA particles, as described above for unmodified HA.

H1N1 influenza virus particles that were purified after sucrose gradient ultracentrifugation were labeled using the same method with a different molar ratio of pHrodo Red (6.125, 12.5, 50, 200 μM). The stain index (mean fluorescence/number of particles) of the virus and the PLGA particles was calculated. Virus particles with a stain index similar to the previously obtained PLGA NPs were chosen and normalized (10^12^/mL) using the NTA method prior to further experiments.

### 2.12. Phagocytosis Analysis

For the detection of phagocytosis kinetics, BMDCs or RAW-Blue cells were seeded in 96-well plates at 2 × 10^5^ cells per well in complete Roswell Park Memorial Institute (RPMI) or DMEM medium, respectively. The next day, RAW-Blue cells were treated with an equal amount of PLGA and virus particles (10^10^ particles per well), and BMDCs were treated with PLGA NPs at 100 μg/mL (which is the equivalent of 10^10^ NPs per well). After the indicated time, cells were washed with PBS, and fluorescence intensity (ex 560 nm, em 585 nm) was measured using a Synergy H4 hybrid reader.

For fluorescent microscopy analysis, BMDCs were cultured in 24-well plates (1 × 10^6^ cells per well) on 13 mm glass coverslips (Corning, New York, NY, USA). Two hours after the addition of PLGA NPs (100 μg/mL), cells were washed twice with ice-cold PBS containing 1% FBS and stained with anti-CD11c FITC antibodies (clone HL3, BD Biosciences, Franklin Lakes, NJ, USA) and DAPI (Sigma-Aldrich, Saint Louis, MO, USA) for 20 min at 4 °C for phagocytosis inhibition. Fluorescent signals from DAPI, FITC, and pHrodo were obtained using Z1 Imager microscope (Carl-Zeiss, Oberkochen, Germany) at 40× magnification.

For flow cytometric analysis, BMDCs were seeded in 96-well plates (2 × 10^5^ cells per well) in RPMI complete medium. The next day, cells were treated for 2 h with PLGA NPs (100 μg/mL), then washed with PBS, and collected using a trypsin-EDTA solution. Cells were stained for 20 min at 4 °C in Staining Buffer with anti-CD11c PE-Cy7 (clone HL3) and anti-MHC class II (I-A/I-E) APC/Cy7 (clone M5/114.15.2) antibodies prior to analysis using FACS AriaIII (all BD biosciences, Franklin Lakes, NJ, USA). Minimum 10.000 CD11c+ MHCII+ cells were analyzed for each sample.

### 2.13. Detection of Maturation Markers on BMDCs

Prior to analysis, BMDCs were seeded in RPMI complete medium at a density of 2 × 10^5^ cells per well on a 24-well plate. Cells were treated with PLGA NPs for 24 h, then washed with PBS and collected using trypsin-EDTA solution. Cells were stained for 20 min at 4 °C in Staining Buffer with anti-CD11c PE-Cy7 (clone HL3), anti-MHC class II (I-A/I-E) APC/Cy7 (clone M5/114.15.2), anti-CD80 PE (clone 16-10A1), anti-CD86 AF700 (clone GL1) antibodies, and analyzed using FACS AriaIII instrument (all from BD biosciences, Franklin Lakes, NJ, USA). Minimum 10.000 CD11c+ MHCII+ cells were analyzed for each sample.

### 2.14. In Vivo and Ex Vivo NF-κB Luminescence Analysis

Analysis was performed as previously described [16]. Briefly, female BALB/c-Tg (Rela-luc)31Xen reporter mice were injected intramuscularly (i.m.) with PLGA NPs or PBS into the thigh muscle (3 mice per group) 3 h prior to analysis. Mice were injected intraperitoneally with a D-luciferin in PBS (3 mg/mouse; Promega, Madison, WI, USA) 2 min before the induction of 2.5% isoflurane anesthesia (Abbot, USA). Luminescence images were collected for 10 s using IVIS Lumina II (Perkin Elmer, USA). NF-κB-dependent luciferase expression was also measured in tissue (lung, kidney, liver, small intestine (referred to as duodenum), colon (referred to as the ascending part), spleen, regionary inguinal lymph node, and thigh muscle (the site of injection)) homogenates of the same mice. Luminescence was measured in normalized tissue homogenates using a Bright-Glo Luciferase Kit (Promega, Madison, WI, USA) and a Synergy H4 reader (BioTek, Winooski, VT, USA).

### 2.15. Animal Studies

C57BL/6 and BALB/c female SPF mice (4–5 weeks old) were purchased from the Animal breeding facility BIBCH RAS (Pushchino, Russia). Transgenic female BALB/c-Tg(Rela-luc)31Xen (4–5 weeks old) mice were purchased from Taconic Biosciences (Rensselaer, NY, USA). Mice were housed in ventilated ISOCage P and N systems (Techniplast, Buguggiate, Italy) for immunological and infection studies, respectively, with free access to autoclaved drinking water and standard chow diet. All of the experimental procedures were made in accordance with the Guide for the Care and Use of Laboratory Animals (NIH Publication #85–23, revised 1996) and approved by the local animal ethics committee of N.F.Gamaleya National Research Center for Epidemiology and Microbiology (protocol #21, 2020).

### 2.16. Immunizations

Female BALB/c and C57BL/6 mice were given three i.m. injections of PLGA NPs into the thigh muscle with a two-week interval between injections. Mice were euthanized by CO_2_ overdose 14 days after the last immunization. Blood from BALB/c and spleens from C57BL/6 were collected for the assessment of humoral and cellular immune responses, respectively. Immunized BALB/c mice were also used for lethal challenge studies.

### 2.17. Analysis of Cellular Immune Response

To assess the cellular responses to antigenic stimulation, splenocytes were collected and purified by density gradient centrifugation (400 *g*, 30 min) using Ficoll 1.09 g/mL (PanEco, Moscow, Russia). T-cell proliferation response was measured as previously described [17] using CellTrace™ carboxyfluorescein diacetate succinimidyl ester (CFSE) Cell Proliferation Kit (Invitrogen, San Diego, CA, USA). Seventy-two hours after addition of A/California/07/2009 HA (Immune Technology Corp, New York, NY, USA), restimulated cells were stained with DAPI (1 µg/mL) to exclude dead cells and anti-CD3 PE-CF594 (clone 145–2C11), anti-CD8 APC (clone 53–6.7), and anti-CD4 APC-Cy7 (clone RM4–5) antibodies for 20 min at 4 °C in Staining Buffer. Proliferating CD4 or CD8 T-lymphocytes were expressed as percent of cells in the final culture that divided at least once (referring to ‘Fraction diluted’ statistic) [18]. Analysis was performed using FACSAriaIII and FACSDiva and FlowJo Software (all from BD Biosciences, Franklin Lakes, NJ, USA). IFN-γ production was measured in cell-free culture supernatants using a bio-plex assay. Minimum 10.000 CD4 + and CD8 + T cells were analyzed for each sample.

### 2.18. Measurement of Antigen-Specific IgG Antibody Titers in Serum Samples from Immunized Mice

Antibody titers of total IgG fraction and IgG1, IgG2a, and IgG2b subtypes in immunized mouse serum specific to an HA antigen were evaluated by ELISA, as previously described [11]. Briefly, 1 μg/mL of HA (Immune Technology Corp, New York, NY, USA) solution was used for coating. Secondary goat anti-mouse antibodies (all from Abcam, Cambridge, UK) diluted 1:5000 in PBS containing 0.05% Tween20 were used for the detection of IgG1, IgG2a, or IgG2b titers. A colorimetric signal was detected after the addition of TMB peroxidase substrate (all from Sigma-Aldrich, Saint Louis, MO, USA) and measured at 450 nm using a Multiscan FC spectrophotometric plate reader (Thermo Fisher Scientific, Waltham, MA, USA) 30 min after the addition of stop solution (4 M H_2_SO_4_).

### 2.19. Hemagglutination Inhibition Assay

The hemagglutinin-binding antibody level in murine serums was assayed by a hemagglutination-inhibition (HI) reaction using chicken erythrocytes [19].

### 2.20. Mouse Infection Studies

A mouse-adapted A/California/07/2009 (H1N1) influenza virus strain was used from a state collection of viruses (ref. #2835) of N. F. Gamaleya National Research Center of Epidemiology and Microbiology, Ministry of Health, Moscow, Russia. The virus was purified and concentrated after centrifugation in a sucrose gradient, and the median lethal dose (LD50) was determined using mice of the same strain and age. Two weeks after the last immunization, BALB/c mice were lightly anaesthetized with ether (Chimmed, Moscow, Russia) and then intranasally received 50 μL of a lethal dose (50LD50) of the virus.

### 2.21. Statistical Analysis

Statistical analysis was performed using GraphPad Prism 8.4 and Excel MS 2016. Residual distributions were checked by Q–Q plots. For antibody titer measurements log2 transformation was done before statistical analysis. Dependent on distribution, one factor and k > 2 groups one-way ANOVA with post-hoc Tukey’s or Dunnett’s test or Kruskal-Wallis test with post-hoc Dunn’s test were used, otherwise two-way or RM ANOVA with post-hoc Tukey’s or Dunnett’s test were performed. Graphs show mean ± SD, box plot display quartiles, or geometric mean ±95% CI. Significance was assumed at *p* < 0.05.

## 3. Results

### 3.1. Preparation and Characterization of Vaccine PLGA NPs Adjuvanted with TLR4/NOD2 Agonists

To evaluate the impact of combined TLR4 and NOD2 stimulation on subunit vaccine efficacy, both immunostimulatory and antigen molecules need to be present in particulate vaccine formulation. The reproducible and tunable technology of NP preparation made up of FDA-approved PLGA polymers presents a versatile tool for the encapsulation of molecules of different weight and origin. Therefore, we prepared PLGA NPs containing hemagglutinin (HA) of the influenza virus A/California/07/2009 (H1N1) as a protective antigen without immunostimulatory molecules (PLGA-HA), with MPLA (PLGA-HA-MPLA) and MDP (PLGA-HA-MDP) alone or in combination (PLGA-HA-MPLA + MDP).

In order to demonstrate the potential beneficial effect of the complex molecular adjuvant, a suboptimal HA dose of 10 ng resulting in minimal (10% survival) animal protection was chosen based on a preliminary lethal influenza infection study (Figure S2).

As NF-κB and AP-1 serves as key transcriptional proinflammatory regulators of immune response activity, we used a readout assay based on NF-κB/AP-1-dependent SEAP reporter RAW-Blue cells expressing both TLR4 and NOD2 to select the ratio of MPLA (1 μg) and MDP (20 μg) that needs to be encapsulated into one dose of PLGA NPs to achieve the potentiation effect (defined as a response to a complex PAMP-based adjuvant that was at least 1.1-fold higher than the sum of the responses induced by each of the individual agonists) on the activity of these transcriptional factors (Figure S3).

Prepared NPs have been characterized and normalized according to several physicochemical parameters known to be involved in vaccine immunogenicity in addition to the antigen/adjuvant content: Particle size [20], surface charge [21], and the presence of residual PVA [22]. (Table 1). Transmission electron microscopy (TEM) analysis was conducted to verify DLS and the NTA particle size analysis of prepared PLGA NPs in addition to identifying the shape of the prepared PLGA NPs (Figure S4).

**Figure 1:**
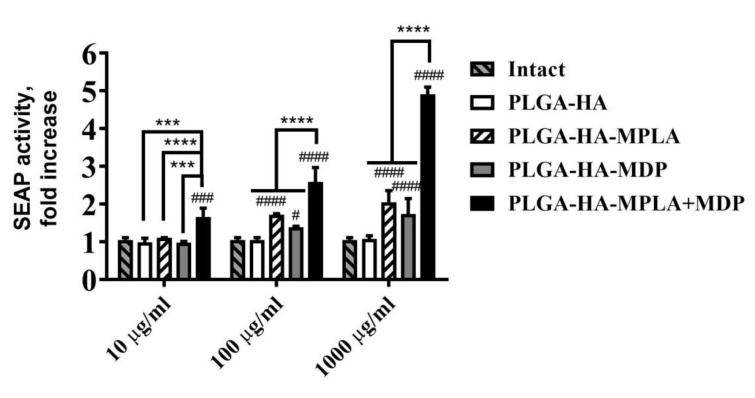
The potentiation of NF-κB/AP-1-dependent secreted embryonic alkaline phosphatase (SEAP) reporter gene expression is not dampened by the encapsulation of monophosphoryl lipid A (MPLA) and muramyldipeptide (MDP) into poly-(D,L-lactic-co-glycolic acid) (PLGA) NPs. The figure represents NF-κB/AP-1-dependent SEAP reporter gene expression in RAW-Blue cells treated for 18 h with PLGA NPs without PRR agonists, with individual PRR agonists or their combination, at the indicated doses (on the x-axis). SEAP activity was measured in cell-free culture supernatants 18 h after the addition of PLGA NPs. Bars represent the mean fold increase ± SD (whiskers) in SEAP activity relative to intact (untreated) cells. Experiment was performed in triplicate. The mean value of untreated cells (= 0.74 ± 0.04 mU/mL) was taken equal to 1 (100%). Significant differences between PLGA NPs with or without pattern-recognition receptor (PRR) agonists are indicated by hashes: ^#^ for *p* < 0.05, ^###^ for *p* < 0.001 or ^####^ for *p* < 0.0001. Significant differences between PLGA-HA-MPLA+MDP and PLGA-HA-MPLA or PLGA-HA-MDP are indicated by asterisks and brackets: *** for *p* < 0.001 or **** for *p* < 0.0001 (two-way ANOVA post-hoc Dunnett’s test).

Prior to infection studies, we first confirmed that the synergistic effects of combined TLR4 and NOD2 stimulation were not affected by the entrapment of PRR agonists into PLGA nanoparticles. For this purpose, we tested the NF-κB/AP-1 stimulating ability of the prepared PLGA vaccine NPs using RAW-Blue reporter cells. The addition of PLGA NPs without immunostimulatory molecules (PLGA-HA) did not result in a significant enhancement of SEAP reporter expression relative to untreated cells at any of the tested doses (Figure 1). The inclusion of MDP into PLGA NPs induced a 1.7-fold NF-κB/AP-dependent SEAP activation (relative to untreated cells) only in the highest dose (1 mg/mL), whereas NPs containing MPLA resulted in significant SEAP reporter expression at both 100 μg/mL (1.7-fold increase over untreated cells) and 1 mg/mL (2.0-fold) doses. The maximum dose-dependent increase of SEAP activity levels—which was significantly higher than for NPs containing individual PRR agonists—was observed using PLGA NPs containing both PRR agonists (PLGA-HA-MPLA+MDP) at 1.7-, 2.6-, and 4.9-fold increases over intact cells at any of the tested doses for 10 μg/mL, 100 μg/mL, and 1 mg/mL, respectively.

Thus, at the lowest (10 μg/mL) dose PLGA NPs containing complex PAMP-based adjuvant results in qualitative changes in NF-κB/AP-1-dependent SEAP expression comparing to control PLGA NPs containing individual PRR agonists. Whereas at the highest dose (1 mg/mL) where all types of PAMP-adjuvanted PLGA NPs induced significant levels of SEAP expression stimulation of both PRRs using PLGA-HA-MPLA + MDP showed clear synergistic effect (4.9/(1.7 + 2.0) = 1.3) on reporter expression. According to obtained data the synergistic activation of NF-κB/AP-1-dependent transcription by the combined stimulation of TLR4 and NOD2 receptors is not dampened by the encapsulation of MPLA and MDP into PLGA NPs.

### 3.2. PLGA NPs Containing a Combination of TLR4 and NOD2 Agonists Are More Effectively Phagocytosed by BMDCs Compared to NPs Containing Individual Agonists

Antigen uptake and the induction of dendritic cell (DC) maturation are the key steps in the formation of adaptive immunity in response to vaccination [23]. It has been reported that individual TLR or NOD2 stimulation promotes phagocytosis [24,25]. However, the effect of the combined stimulation of TLR4 and NOD2 using PLGA NPs containing a complex adjuvant on phagocytosis efficacy was not investigated.

**Figure 2:**
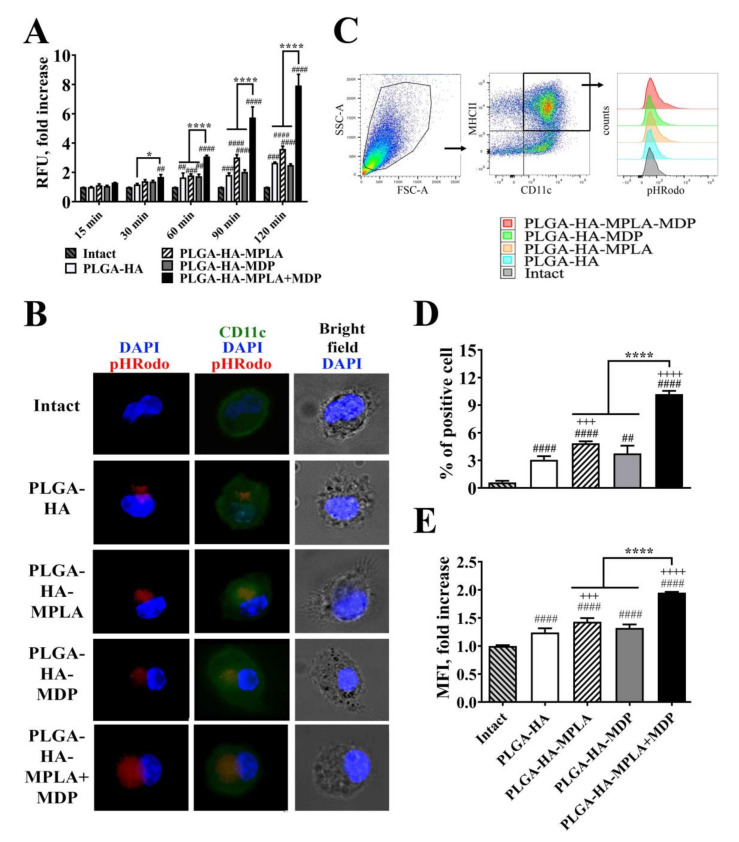
PLGA NPs containing both MPLA and MDP are more effectively phagocytosed by bone marrow-derived dendritic cells (BMDCs) compared to NPs containing individual PRR agonists. (**A**) A time course of phagocytosis quantification by BMDCs of PLGA NPs containing pHRodo-stained hemagglutinin (HA). Immature BMDCs isolated from naïve C57BL/6 mice were seeded in a 96-well plate at 2 × 10^5^ cells per well in complete RPMI medium. The next day, PLGA-HA, PLGA-HA-MPLA, PLGA-HA-MDP, and PLGA-HA-MDP-MPLA formulations produced with pHRodo-labeled hemagglutinin were added in a 100 μg/mL final concentration. The medium was removed at the indicated time points, BMDCs were washed with PBS, and the integral fluorescence of each well was determined using a Biotek microplate reader. Each bar represents the mean fold increase ± SD (whiskers) in relative fluorescence units (RFU) over intact (untreated) cells. The mean value of untreated cells (= 1088 ± 10 RFU) was taken equal to 1 (100%). Experiment was performed in triplicate and repeated twice. (**B**) Representative fluorescence microscopy images of BMDCs treated with pHRodo-stained PLGA NPs for 120 min. BMDCs were stained with anti-CD11c antibodies (green). The cell nucleus was visualized with DAPI (blue). Phagocytosed PLGA NPs were specifically visualized by pHRodo (red) fluorescence. Scale bar represents 20 μm. (**C**) Representative gating strategy used to assess the phagocytic activity of BMDCs. BMDCs were initially gated in an forward versus side scatter (FSC vs. SSC) dot plot and then identified as double positive (MHCII+ CD11c+) population. Phagocytic activity of BMDCs was determined by (**D**) % of pHRodo-positive BMDCs as well as (**E**) fold increase in mean fluorescence intensity (MFI) of total MHCII+ CD11c+ cells relative to untreated cells 120 min after the addition of PLGA NPs. Mean value of untreated cells (= 749.5 ± 9.2 RFU) was taken equal to 1 (100%). Each bar represents the mean ± SD from three independent experiments, each performed in triplicate. Significant differences between PLGA NPs and intact cells are indicated by hashes: ^##^ for *p* < 0.01, ^###^ for *p* < 0.001, or ^####^ for *p* < 0.0001. Significant differences between PLGA NPs with or without PRR agonists are indicated by plus signs: +++ for *p* < 0.001 or ++++ for *p* < 0.0001. Significant differences between PLGA-HA-MPLA+MDP and PLGA-HA-MPLA or PLGA-HA-MDP are indicated by asterisks and brackets: * for *p* < 0.05, or **** for *p* < 0.0001. P-values were calculated using two-way RM ANOVA post-hoc Dunnett’s test (**A**) and one-way ANOVA post-hoc Tukey’s test (**D,E**).

For this experiment, we prepared NPs containing hemagglutinin labeled with pH-dependent fluorescent pHrodo dye used as a specific sensor of antigen entrapment into phagosomes. Prepared vaccine NPs were added to immature murine bone marrow-derived dendritic cells (BMDCs). First, we examined the time-dependent effects of individual and combined TLR4 and NOD2 stimulation on the phagocytosis of vaccine particles containing PRR agonists (Figure 2A). The addition of vaccine NPs adjuvanted with individual PRR agonists resulted in a significant increase in fluorescence over intact cells starting only from the 60 min time point (a 1.8-fold increase for PLGA-HA-MPLA and a 1.7-fold increase for PLGA-HA-MDP), whereas PLGA-HA-MPLA + MDP-treated cells became remarkably fluorescent starting from the 15 min time point. At all time points (15, 30, 60, 90, and 120 min), cells treated with PLGA-HA-MPLA + MDP had the highest fluorescent signal among the studied groups. The maximum values compared to untreated cells were observed 120 min after the addition of PLGA-HA-MPLA (3.6-fold), PLGA-HA-MDP (2.5-fold), and PLGA-HA-MPLA+MDP (7.9-fold). This time point was used for a detailed investigation of observed differences in vaccine particle internalization by fluorescence microscopy and flow cytometry analysis.

For phagocytosis visualization, BMDCs were treated with different PLGA NPs for 120 min, extensively washed, stained with CD11c antibodies (green), and analyzed by the intensity of red fluorescence corresponding to the quantity of pHRodo-stained HA entrapped in phagosomes (red) (Figure 2B). At the chosen time point, only a small amount of vaccine particles containing no PRR agonists was found in the cytoplasm of the cells. The inclusion of MDP or MPLA individually in the PLGA NPs resulted in increased particle internalization, as visualized by red fluorescence. However, the highest levels of internalized NPs were observed in cells treated with the vaccine containing both PRR agonists.

Flow cytometry analysis was used in order to obtain quantitative characteristics of the phagocytosis process, namely the percentage of phagocytizing (pHrodo-positive) cells and the number of internalized particles per cell (indicated by mean fluorescence intensity, MFI) (Figure 2C–E). Compared to PLGA-HA (3.1% relative to intact cells), the inclusion of individual MDP or MPLA in vaccine NPs resulted in a greater percentage of cells involved in phagocytosis (3.8% and 4.9% of BMDCs were pHrodo-positive, respectively). The highest percentage was observed in BMDCs incubated with PLGA NPs containing both MDP and MPLA (10.2% of cells were pHrodo-positive). The last group was also characterized by a maximal number of internalized particles per cell (2.0-fold MFI increase over intact cells) compared to PLGA-HA-MPLA (1.5-fold), PLGA-HA-MDP (1.3-fold), and PLGA-HA (1.3-fold) NPs.

Thus, this study is the first report to demonstrate that the inclusion of complex adjuvants containing TLR4 and NOD2 agonists in PLGA NPs has a promoting effect on different aspects of vaccine phagocytosis by BMDCs.

### 3.3. Comparison of Phagocytosis Rate and Immunostimulatory Activity of PLGA NPs and H1N1 Influenza Viral Particles In Vitro

**Figure 3:**
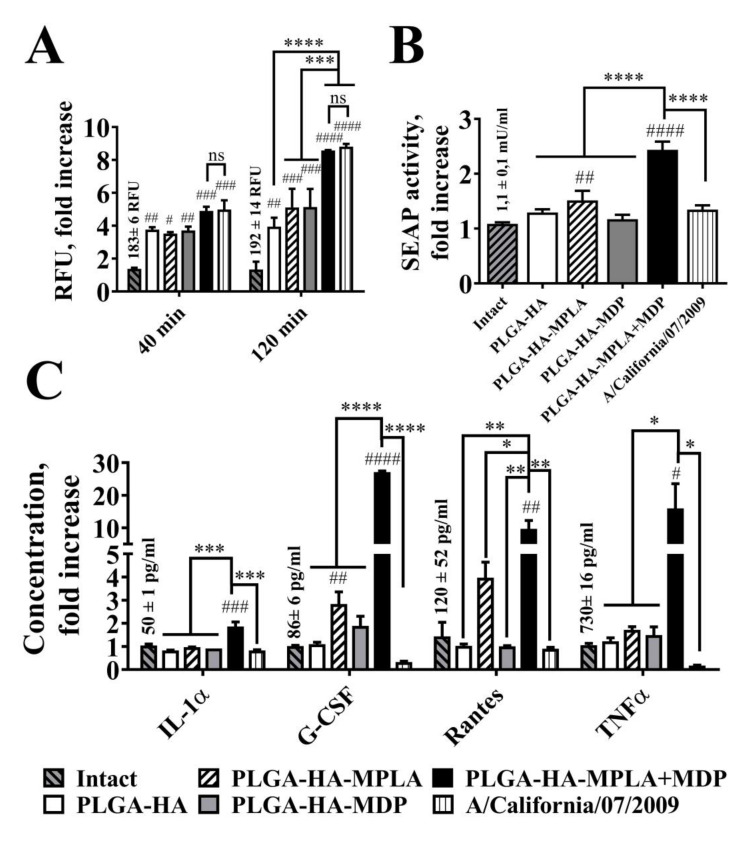
Phagocytosis rate and cell response after the addition of equal amounts of PLGA NPs and H1N1 influenza viral particles to RAW-Blue cells. (**A**) Phagocytosis quantification of PLGA NPs and H1N1 influenza viral particles. RAW-Blue cells were seeded in 96-well plate at 2 × 10^5^ cells per well in complete RPMI medium. The next day, normalized to 10^10^ particles/well PLGA (roughly equal to 100µg/ml) and virus particles were added to the cells. The medium was removed at the indicated time points, the cells were washed with PBS, and the integral fluorescence (RFU) of each well was determined using a Biotek reader. Mean values (indicated above the bars) of intact cells were taken equal to 1 (100%). Data represent the mean (*n* = 3) fold increase over intact cells ± SD. (**B**) NF-κB/AP-1-dependent SEAP reporter gene expression in cell-free culture supernatants from RAW-Blue cells treated for 18 h with PLGA and with virus particles normalized by quantity (10^10^ particles/well). Mean values (indicated above the bars) of intact cells were taken equal to 1 (100%). Data represent the mean (*n* = 3) fold increase over intact cells ± SD. (**C**) Cytokine levels were measured in cell-free culture supernatants from the same wells using bead-based immunoassay. Bars represent mean fold increase ± SD (whiskers) over intact group. Values (mean concentrations ± SD are indicated above the bars) of intact groups were taken equal to 1 (100%). Significant differences between treated and intact cells are indicated by hashes: ^#^ for *p* < 0.05, ^##^ for *p* < 0.01, ^###^ for *p* < 0.001, or ^####^ for *p* < 0.0001. Statistically significant differences are shown among groups as indicated by asterisks and brackets: * for *p* < 0.05, ** for *p* < 0.01, *** for *p* < 0.001, or **** for *p* < 0.0001. *p*-values were calculated using two-way ANOVA post-hoc Tukey’s test (**A**) and one-way ANOVA post-hoc Tukey’s test (**B**,**C**).

It is considered that live attenuated vaccines possess higher immunogenic properties compared to subunit vaccines. To evaluate whether adjuvantation with a combination of TLR4 and NOD2 agonists could elevate immunogenicity of subunit vaccines comparable to ones based on whole pathogens, we focused on the earliest events of host–pathogen interaction (phagocytosis, activation of proinflammatory transcription factors and cytokine expression) crucial for further adaptive immune response, but where other mechanisms specific for live attenuated vaccines (e.g., antigen propagation due to pathogen replication) are disregarded. We used a mouse adapted A/California/07/2009 (H1N1) virus as an example of LAIV. It should also be noted that while the size of the particles could affect the immunogenicity of particulate vaccines, the influenza virus could be an adequate counterpart for prepared PLGA NPs with similar dimensions, i.e., within 80–120 nm [26]. We determined the size distribution of the obtained samples of PLGA NPs and H1N1 influenza virus using the NTA method (Figure S5). Subunit and whole virus vaccines showed similar statistically indistinguishable sizes (161 ± 55 nm for PLGA-HA-MPLA + MDP and 91 ± 64 nm for influenza virus samples), which allow us to speculate that further effects will only be a result of differences in particle content. In the next step, in order to measure the quantity of entrapped particles into phagosomes, we stained viral particles with pHrodo dye and obtained virus sample with a stain index (mean fluorescence/number of particles) similar to that previously obtained for the PLGA NPs (2.6 × 10^−9^ for PLGA-HA-MPLA+MDP and 3.5 × 10^−9^ for influenza virus samples). Lastly, we normalized the numbers of particles in both samples using the NTA method (10^10^ particles per well for PLGA-HA-MPLA+MDP and influenza virus samples) before addition to RAW-Blue cells. At all measured time points (40 and 120 min), the addition of virus particles and PLGA-HA-MPLA+MDP showed significantly higher levels of specific fluorescent signals compared to PLGA NPs containing individual PRR agonists (Figure 3A). A similar (statistically not significant) maximal fluorescence was registered after the addition of PLGA NPs containing both MDP and MPLA molecules and influenza virus particles. It is also important to note that the addition of PLGA-HA-MPLA + MDP (2.4-fold increase over intact cells) resulted in higher NF-κB/AP-1-dependent SEAP reporter gene expression in RAW-Blue cells compared to an equal number of virus particles (1.3-fold increase) (Figure 3B). The evaluation of cytokine production levels showed that the addition of PLGA-HA-MPLA + MDP induced a maximal production of IL-1α (1.9-fold increases over intact cells), G-CSF (27-fold increase), RANTES (9.7-fold increase), and TNFα (15.9-fold increase) (Figure 3C), whereas cells treated with influenza virus particles responded with decreased levels of G-CSF and TNFα over intact cells, which is in line with previous data describing the prominent ability of the H1N1 strain to inhibit cytokine responses in human macrophages [27]. Along with the absence of the toxic effects of influenza virus on RAW-Blue cells, the obtained results show that a complex adjuvant is able to increase the phagocytosis of subunit vaccine particles equal to control particles made of whole pathogens with even greater effects on further cell response.

### 3.4. PLGA NPs Containing a Combination of TLR4 and NOD2 Agonists Significantly Enhance Maturation of BMDC Compared to NPs Containing Individual PRR Agonists

After antigen engulfment, the further activation of antigen-presenting cells (APCs) mediates effective induction of the adaptive immune response. According to this, we evaluated the expression of costimulatory molecules associated with DC maturation: CD80, CD86, and cytokine production profiles in response to the addition of PLGA NPs containing individual TLR4 and NOD2 agonists and their combination.

**Figure 4:**
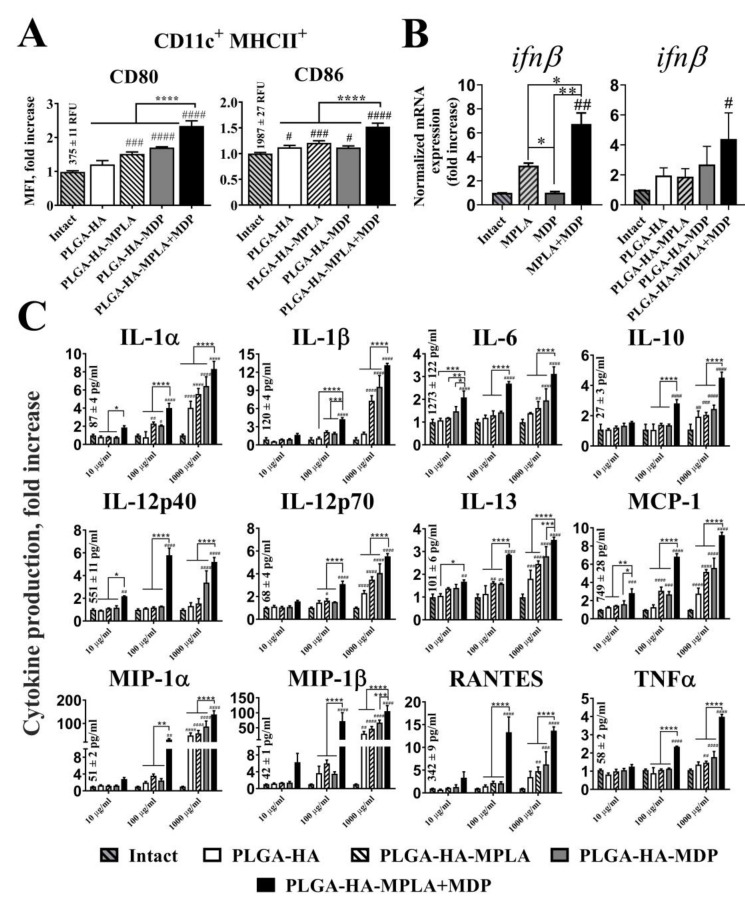
PLGA NPs containing both MPLA and MDP significantly enhance the cytokine response of BMDCs as well as the expression of maturation markers compared to NPs containing individual PRR agonists. (**A**) The expression of the maturation markers, CD80 and CD86, of BMDCs treated with PLGA NPs. MFI (indicating expression level) fold increase values relative to intact (untreated) cells ± SD are shown. Mean values (indicated above the bars) of intact cells were taken equal to 1 (100%). Experiment was performed in triplicate and repeated twice. (**B**) qRT-PCR analysis of *ifnβ* expression in BMDCs following stimulation with soluble MPLA (1μg/mL) and MDP (20μg/mL) molecules as well as PLGA NPs (100μg/mL) for 72 h. The data were normalized to *gapdh* in each sample and presented as mean fold change (± SD) relative to intact cells (taken as 1). Minimum three replicates were used in each group. (**C**) Cytokine levels in cell-free culture supernatants from the BMDCs treated with PLGA NPs in indicated doses. Data represent the mean (*n* = 5) fold increase over intact cells ± SD. Mean values (mean concentrations ± SD are indicated above the bars) in intact cells were taken equal to 1 (100%). Significant differences between PLGA NPs and intact cells are indicated by hashes: ^#^ for *p* < 0.05, ^##^ for *p* < 0.01, ^###^ for *p* < 0.001, or ^####^ for *p* < 0.0001. Significant differences between PLGA-HA-MPLA+MDP and PLGA-HA-MPLA or PLGA-HA-MDP are indicated by asterisks and brackets: * for *p* < 0.05, ** for *p* < 0.01, *** for *p* < 0.001, or **** for *p* < 0.0001. *p*-values were calculated using one-way ANOVA post-hoc Tukey’s test (**A,B**) and two-way ANOVA with Dunnett’s post hoc test (**C**).

The expression levels of CD80 and CD86 were measured 18 h after the addition of PLGA NPs to immature BMDCs using flow cytometry analysis. The addition of PLGA NPs containing individual PRR agonists (PLGA-HA-MPLA and PLGA-HA-MDP) to BMDCs increased the levels of CD80 (1.5-and 1.7-fold increases over intact cells, respectively) and CD86 (1.2-and 1.1-fold increases, respectively) expression, compared to untreated cells (Figure 4A). Along with this, the maximal levels of CD80 (2.4-fold) and CD86 (1.5-fold) maturation markers were observed after the addition of PLGA NPs containing both PRR agonists (PLGA-HA-MPLA + MDP).

Cytokine response of BMDCs after PLGA NPs addition was studied using quantitative real time PCR (qRT-PCR) analysis (IFNβ mRNA expression) and bead-based 23-plex assay. It is known that stimulation of TLR4 and NOD2 receptors play a critical role in the type I interferon response [28]. Here, we analyzed expression of IFNβ mRNA in BMDCs in the context of combined stimulation of TLR4 and NOD2 receptors (Figure 4B). Stimulation of both PRRs using soluble agonists resulted in significantly higher level of IFNβ response (mean 6.7-fold increase over intact cells) compared to individual TLR4 (3.4-fold) or NOD2 (1.1-fold) stimulation. Importantly, similar results were obtained using combination of PRR agonists encapsulated into PLGA NPs. Maximal levels of IFNβ response were registered in BMDCs treated with PLGA-HA-MPLA + MDP (4.4-fold) compared to control PLGA-HA-MPLA (1.9-fold) and PLGA-HA-MDP (2.7-fold) formulations.

Concentration of other 23 cytokines was measured in cell-free culture supernatants collected from BMDCs incubated with the PLGA NPs at different concentrations (from 10 μg/mL up to 1 mg/mL) for 24 h (Figure 4C). At the maximum tested concentration (1 mg/mL), the addition of PLGA-HA-MPLA or PLGA-HA-MDP to cells resulted in the enhanced production of 6 out of 23 of the measured cytokines in comparison to intact cells, namely IL-1α (5.6- and 6.5-fold increase, respectively), IL-1β (7.3- and 9.6-fold), IL-12p70 (3.5- and 4.1-fold), IL-13 (1.6- and 1.5-fold), MCP-1 (5.2- and 5.6-fold), and MIP-1β (47.1- and 67.3-fold). The addition of PLGA NPs containing both PRR agonists expanded the number of registered cytokines with elevated expression and raised cytokine concentration levels in comparison to control groups. Thus, the addition of PLGA-HA-MPLA+MDP at 1 mg/mL, compared to control formulations, induced 12 out of the 23 measured cytokines: IL-1α (8.3-fold), IL-1β (13.2-fold), IL-12p70 (5.5-fold), IL-13 (3.5-fold), MCP-1 (9.2-fold), MIP-1β (105.5-fold). (including IL-6 (3.1-fold), IL-10 (4.5-fold), IL-12p40 (5.2-fold), MIP-1α (138.8-fold), RANTES (13.7-fold), and TNFα (4.0-fold).

The maximal potentiation effect over PLGA NPs with individual PRR agonists was present using a suboptimal concentration (100μg/mL). At this concentration, expression levels of 11 cytokines after PLGA-HA-MPLA + MDP addition were greater than the sum of the fold induction observed after the addition of PLGA NPs containing individual PRR agonists: IL-1α (1.2-fold increase over the sum of the fold induction observed after stimulation of either TLR4 or NOD2 alone), IL-1β (1.4-fold increase), IL-12p70 (1.4-fold increase), MCP-1 (1.4-fold increase), IL-6 (1.6-fold increase), IL-10 (1.6-fold increase), IL-12p40 (4.0-fold increase), RANTES (4.0-fold increase), MIP-1α (6.1-fold increase), MIP-1β (8.4-fold increase), and TNFα (2.0-fold increase).

We also compared cytokine responses of BMDC treated with PLGA-HA-MPLA+MDP (200μg/mL) versus equally mixed PLGA NPs containing individual PRR agonists PLGA-HA-MPLA and PLGA-HA-MDP (200μg/mL each) (Figure S6). Surprisingly, in a context of equal amounts of immunostimulatory molecules vaccine formulation containing both PRR agonists resulted in greater production of number of cytokines (IL-10, IL-13, MCP-1, TNFα) compared to vaccine particles containing separated PRR agonists.

Overall, these results demonstrate that the complex vaccine adjuvant consisting of MPLA+MDP provokes significantly higher levels of BMDC maturation and activation in comparison to adjuvants based on individual PRR agonists.

### 3.5. PLGA NPs Containing a Combination of TLR4 and NOD2 Result in Enhanced NF-κB Activity Levels after i.m. Administration in Mice

To determine whether the enhanced in vitro activation of NF-κB after the addition of PLGA NPs to the cells containing a combination of PRR agonists also occurs in vivo, we used BALB/c-Tg(Rela-luc)31Xen reporter mice carrying an NF-κB-dependent luciferase reporter gene.

NF-κB-Luc transgenic mice were i.m. injected in the thigh muscle with 100 μL of prepared PLGA NPs (1 mg/mouse). Control mice were i.m. injected with 100 μL of PBS. NF-κB activity was detected by the live imaging of mice 3 h after the administration of PLGA NPs.

**Figure 5:**
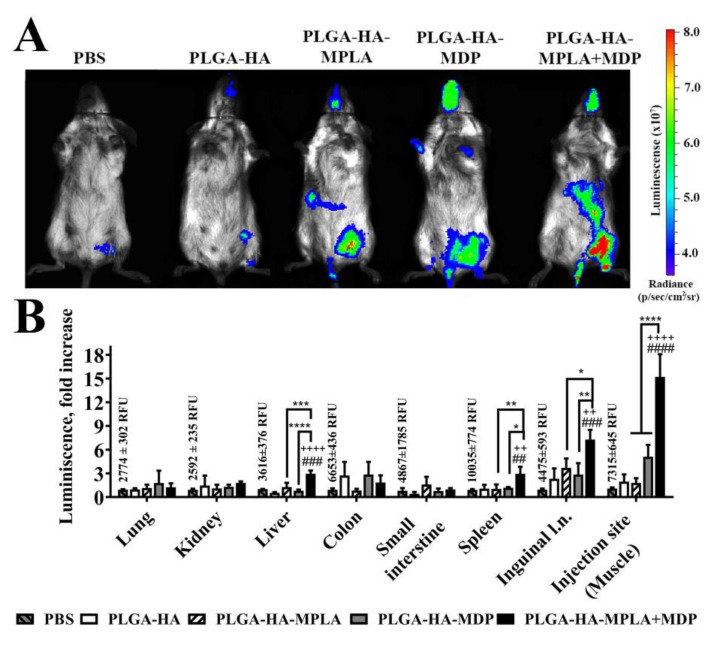
PLGA NPs containing both MPLA and MDP induce stronger NF-κB transcriptional response in BALB/c-Tg (Rela-luc) 31Xen transgenic mice than NPs with individual PRR agonists. (**A**) Representative bioluminescent pseudocolored images of NF-κB-luciferase activity in live NF-κB-Luc transgenic mice 3 h after i.m. injection of PBS, PLGA-HA, PLGA-HA-MPLA, PLGA-HA-MDP, or PLGA-HA-MPLA + MDP (all in 1 mg/mouse in 100 μL). Prior to imaging, mice received an i.p. injection of luciferin followed by anesthesia. Non-immunized control mice received PBS. The intensity of bioluminescence (i.e., NF- κB activity) is shown in color according to the scale on the right. Two additional mice in each group gave similar results. (**B**) NF-κB-dependent bioluminescence in tissue homogenates prepared from NF-κB-Luc transgenic mice at the same time point after the injection of PLGA NPs. Control mice were injected with PBS. Values of control group (mean fluorescence intensities ± SD are indicated above the bars) were taken equal to 1 (100%). Results are expressed as the fold increase in relative luminescence units over PBS-treated control animals. Each bar represents the mean of three mice per group ± SD (error bars). Significant differences between PLGA NPs and PBS treated mice are indicated by hashes: ^##^ for *p* < 0.01, ^###^ for *p* < 0.001, or ^####^ for *p* < 0.0001. Significant differences between PLGA NPs with or without PRR agonists are indicated by plus signs: ++ for *p* < 0.01 or ++++ for *p* < 0.0001. Significant differences between PLGA-HA-MPLA + MDP and PLGA-HA-MPLA or PLGA-HA-MDP are indicated by asterisks and brackets: * for *p* < 0.05, ** for *p* < 0.01, *** for *p* < 0.001, or **** for *p* < 0.0001. P-values were calculated using one-way ANOVA post-hoc Tukey’s test.

The obtained data show that PLGA-HA results in minimal NF-κB elevation at the site of injection compared to PBS-treated mice (Figure 5A). The inclusion of individual PRR agonists into PLGA NPs results in a significantly increased area and an NF-κB-dependent luminescence level at the site of injection compared to NPs without any PRR agonist. The injection of PLGA-HA-MPLA + MDP resulted in the highest luminescence level at the site of injection with distribution to the abdominal region. To identify organs and tissues involved in immune response after immunization with PLGA NPs containing a complex adjuvant, we measured luminescence in the protein lysates of thigh muscles (injection site) and of inguinal lymph nodes (closest to the injection site), lungs, kidneys, liver, colon, small intestine, and spleen. PLGA NPs without PRR agonists did not result in a significant increase in the NF-κB-dependent luminescence signal of any organ or tissue (Figure 5B). Interestingly, vaccination with PLGA-HA-MDP and PLGA-HA-MPLA resulted in different activation profiles. PLGA-HA-MDP significantly increased NF-κB-dependent luminescence at the site of injection (5.1-fold increase), whereas PLGA-HA-MPLA induced significantly higher levels in inguinal lymph nodes (3.7-fold increase) over PBS-treated mice. PLGA NPs containing both PRR agonists resulted in maximal levels of NF-κB activation at both sites of injection (15.2-fold increase over PBS-treated mice) and inguinal lymph nodes (7.3-fold increase) as well as in liver (3.0-fold increase) and spleen (3.0-fold increase), which were not affected after immunization with PLGA-HA-MDP and PLGA-HA-MPLA NPs. 

In order to investigate that observed elevation of NF-κB-dependent luminescence in mice is not a result of systemic toxicity of PLGA NPs, we have conducted safety study experiments using most biologically active PLGA-HA-MPLA + MDP NPs in previously used dose (1mg) and twice higher. None of the doses did not result in significant differences in complete blood count (CBC) and routine biochemistry parameters comparing to control PBS-treated mice (Table S1, Table S2, Table S3). 

The obtained results indicate that the registered in vitro effect of NF-κB potentiation using solubilized TLR4 and NOD2 agonists is also present in vivo if such a combination of PRR agonists is used in the context of vaccine particles.

### 3.6. PLGA NPs Containing a Combination of TLR4 and NOD2 Result in Enhanced Local Cytokine Production after i.m. Administration in Mice

**Figure 6:**
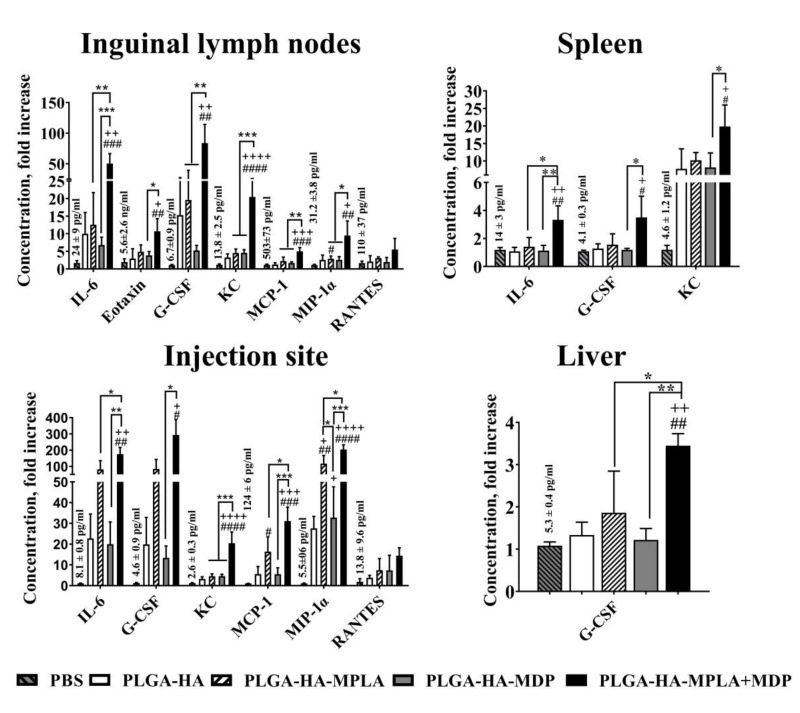
PLGA NPs containing both MPLA and MDP induce a stronger local cytokine response in mice than NPs containing individual PRR agonists. BALB/c mice were intramuscularly injected with 1 mg/dose of PLGA-HA, PLGA-HA-MPLA, PLGA-HA-MDP, or PLGA-HA-MPLA + MDP. Non-immunized control mice received PBS. Three hours later, inguinal lymph nodes, the liver, the spleen, and the thigh muscle (corresponding to the injection site) were isolated. Cytokine levels were measured in tissue homogenates using bead-based immunoassay. Control mice were injected with PBS. Results are expressed as the mean fold change in cytokine concentration relative to the mean concentration in PBS-treated control animals. Values of control group (mean concentrations ± SD are indicated above the bars) were taken equal to 1 (100%). Each point represents the mean of three mice per group ± SD (error bars). Significant differences between PLGA NPs and intact cells are indicated by hashes: ^#^ for *p* < 0.05, ^##^ for *p* < 0.01, ^###^ for *p* < 0.001, or ^####^ for *p* < 0.0001. Significant differences between PLGA NPs with or without PRR agonists are indicated by plus: + for *p* < 0.05, ++ for *p* < 0.01, +++ for *p* < 0.001, or ++++ for *p* < 0.0001. Significant differences between PLGA-HA-MPLA+MDP and PLGA-HA-MPLA or PLGA-HA-MDP are indicated by asterisks and brackets: * for *p* < 0.05, ** for *p* < 0.01, or *** for *p* < 0.001. P-values were calculated using one-way ANOVA post-hoc Tukey’s test.

To characterize the early immune outcome of potentiated levels of NF-κB after immunization with prepared PLGA NPs, we measured the concentration of cytokines at the site of injection and in organs where potentiated NF-κB activity has been registered. For this purpose, BALB /c mice (5 per group) were immunized with PLGA NPs (1 mg/mouse). Twenty-four hours after injection, the concentrations of 23 cytokines were measured in thigh muscle as well as liver, spleen, and inguinal lymph node homogenates (Figure 6). The broadest cytokine expression profile was detected in inguinal lymph nodes. Here, PLGA-HA-MPLA + MDP induced the expression of 7 out of 23 cytokines: IL-6 (50.4-fold), eotaxin (10.7-fold), G-CSF (83.8-fold), KC (20.5-fold), MCP-1 (5.0-fold), MIP-1α (9.5-fold), and RANTES (5.5-fold) over PBS-treated mice. Vaccination with PLGA-HA-MDP induced the production of 3 of the 23 measured cytokines: IL-6 (6.8-fold), G-CSF (5.2-fold), and KC (4.6-fold), whereas only KC (4.5-fold) and MIP-1α (2.9-fold) were elevated after PLGA-HA-MPLA immunization. At the injection site, the number of cytokines with elevated levels was slightly lower than in the lymph nodes (6 out of 23) after the injection of PLGA-HA-MPLA + MDP: IL-6 (174.4-fold), G-CSF (293.1-fold), KC (20.5-fold), MCP-1 (31,1-fold), MIP-1α (204.0-fold), and RANTES (14.5-fold). The injection of PLGA-HA-MPLA resulted in elevated levels of KC (4.5-fold) and MIP-1α (2.9-fold). PLGA-HA-MDP induced increased expression of IL-6 (6.8-fold), G-CSF (5.2-fold), and KC (4.6-fold) compared to PBS treated mice. A much weaker response was observed in the spleen. The production of 3 out of 23 cytokines was significantly elevated after the injection of PLGA-HA-MPLA + MDP: IL-6 (3.3-fold), G-CSF (3.5-fold), and KC (19.9-fold). Only KC levels were elevated in the spleen after PLGA-HA-MPLA (10.2-fold) or PLGA-HA-MDP (7.8-fold) immunization. The dimmest effect on cytokine production was observed in the liver. Only G-CSF was significantly elevated after the immunization with PLGA NPs containing both PRR agonists: 3.5-fold increase over the PBS-treated group.

These results indicate that the adjuvantation of PLGA NPs with a combination of TLR4 and NOD2 agonists not only increases the levels of cytokine production but also broadens the number of organs and tissues involved in the immune response after subunit vaccine administration.

### 3.7. Vaccination with PLGA NPs Containing a Combination of TLR4 and NOD2 Agonists Induces Stronger Antigen-Specific CD4 T-Cell Response in Mice than PLGA NPs Containing Individual PRR Agonists

The observed synergistic effects of combined TLR4 and NOD2 stimulation both in vitro and in vivo suggest that PLGA NPs with a combination of TLR4 and NOD2 agonists will elicit a much stronger adaptive immune response upon vaccination.

**Figure 7:**
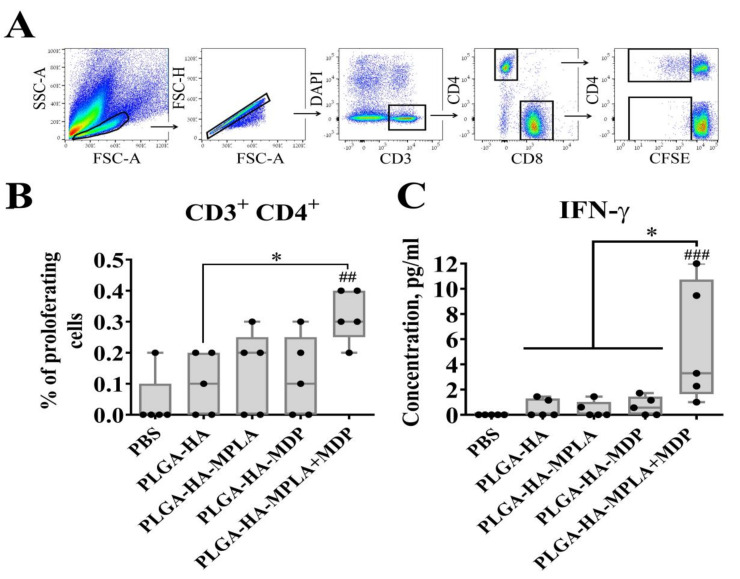
PLGA NPs containing both MPLA and MDP induce stronger antigen-specific CD4+ T-cell response in mice than NPs containing individual PRR agonists. C57BL/6 mice (*n* = 5/group) were i.m. immunized three times with PLGA NPs: PLGA-HA, PLGA-HA-MPLA, PLGA-HA-MDP, and PLGA-HA-MPLA + MDP with a two-week interval. Non-immunized control mice received PBS. Splenocytes were harvested from mice 14 days after the last immunization. Splenocytes were stained with CFSE, treated with whole hemagglutinin (1 μg/mL). 72 h after cell were harvested for cellular proliferation analysis, cell culture was collected for IFN-γ detection. (**A**) Gating strategy for assessment of proliferating T cells in culture. Single lymphocytes were gated using the forward and side scatter characteristics. Live (DAPI negative) CD3 + T cells were then obtained from the single cells gate. CFSE^dim^ cells were gated in each CD3 + CD4+ as well as CD3 + CD8+ populations. (**B**) % of proliferating CD4 + T-cells (referred to fraction diluted) in response to hemagglutinin restimulation. (**C**) IFN-γ levels in supernatants from HA-stimulated spleen cells, determined by a bio-plex assay 72 h after restimulation. Boxes show interquartile range, whiskers show range, and horizontal lines represent median values. Dots show individual data points. Significant differences (*p* < 0.05) between PLGA NPs and PBS-treated mice are indicated by hashes: ^##^ *p* < 0.01, or ^###^ *p* < 0.001. Significant differences between PLGA-HA-MPLA+MDP and the NPs with individual PRR agonists (PLGA-HA-MDP or PLGA-HA-MPLA) or without PRR agonists (PLGA-HA) are indicated by asterisks and brackets (* *p* < 0.05). P-values were calculated using Kruskal–Wallis post-hoc Dunn’s test.

To test this in terms of cell-mediated immune response, we immunized mice with prepared PLGA NPs (three intramuscular injections were given with two week intervals, 5 mice/group), and splenocytes were then isolated for analysis two weeks after the last immunization. CD4+ and CD8+ T-cell responses were detected by a CFSE fluorescence proliferation assay (Figure 7A,B) and IFNγ production 72 h after hemagglutinin restimulation in vitro (Figure 7C). The obtained data show that HA-stimulated T-cell expansion in all experimental groups was limited to CD4+ proliferating T-cells. (The insignificant differences in the percentage of CFSE^dim^ CD8+ T cells are not shown). The encapsulation of individual TLR4 and NOD2 agonists in vaccine PLGA NPs did not result in a significant increase in proliferating CD4 T-cells (0.14% and 0.12%, respectively) compared to a group immunized with PLGA-HA NPs without immunostimulatory molecules (0.10%). The only statistically significant difference was seen in the mice immunized with PLGA-HA-MPLA + MDP (0.32% CD4+ T-cells) compared to PBS- and PLGA-HA-treated mice.

Consistent with the T-cell proliferative response, IFN-γ production was found to be maximal in the group of mice vaccinated with PLGA-HA-MPLA + MDP (a mean concentration of 5.1 ng/mL) compared to PBS- and PLGA-HA-treated mice (Figure 7C). Mice immunized with PLGA-HA-MDP (0.5 ng/mL) or PLGA-HA-MPLA (0.4 ng/mL) did not result in significantly elevated levels of IFN-γ production compared to the PLGA-HA-immunized group (a mean concentration of 0.5 ng/mL).

Thus, a complex adjuvant based on a combination of TLR4 and NOD2 agonists used in PLGA NPs significantly increased the magnitude of T-cell mediate immunity in comparison to individual PRR agonists.

### 3.8. PLGA NPs Containing a Combination of TLR4 and NOD2 Agonists Lead to a Stronger Hemagglutinin-Specific Antibody Response in Mice than NPs Containing Individual PRR Agonists

To evaluate the effects of individual or combined stimulation of TLR4 and NOD2 receptors on humoral adaptive immune response, we immunized mice using the same scheme (three intramuscular injections with two week intervals, *n* = 5 mice/group) with PLGA NPs and determined titers of HA-specific IgG and hemagglutination-inhibiting (HI) antibodies in blood serum 14 days after the last immunization. 

**Figure 8:**
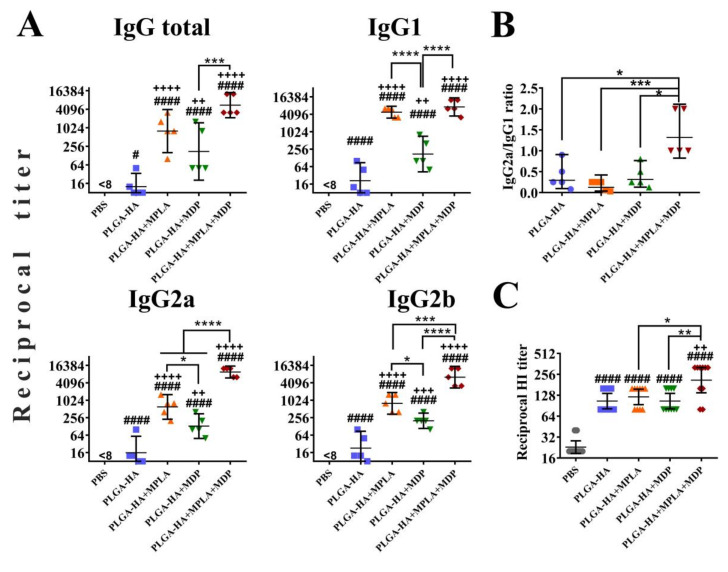
PLGA NPs containing both MPLA and MDP lead to a stronger humoral adaptive response in mice than NPs containing individual PRR agonists. BALB/c mice (*n* = 5/group) were i.m. immunized three times with PLGA NPs: PLGA-HA, PLGA-HA-MPLA, PLGA-HA-MDP, and PLGA-HA-MDP-MPLA. Non-immunized control mice received PBS. Blood was collected from mice 14 days after the last immunization for serum preparation. The geometric mean for 5 mice/group with 95%CI is shown. (**A**) The reciprocal titers of hemagglutinin-specific total IgG antibodies and IgG1, IgG2a, IgG2b, and IgG2c subtypes were detected in serum by ELISA. The geometric mean for 5 mice/group with 95% CI is shown. (**B**) Ratios of IgG2a/IgG1 isotype HA-specific antibodies. (**C**) The reciprocal titers of hemagglutination inhibition (HI) antibodies against the H1N1 virus in the sera of mice immunized with PLGA-HA, PLGA-HA-MPLA, PLGA-HA-MDP, and PLGA-HA-MDP-MPLA. Significant differences between PLGA NPs and intact cells are indicated by hashes: ^#^ for *p* < 0.05 or ^####^ for *p* < 0.0001. Significant differences between PLGA NPs with or without PRR agonists are indicated by plus: ++ for *p* < 0.01, +++ for *p* < 0.001, or ++++ for *p* < 0.0001. Significant differences between PLGA-HA-MPLA+MDP and the NPs with individual PRR agonists (PLGA-HA-MDP or PLGA-HA-MPLA) are indicated by asterisks and brackets: * for *p* < 0.05, ** for *p* < 0.01, *** for *p* < 0.001, or **** for *p* < 0.0001. *p*-values were calculated using one-way ANOVA post-hoc Tukey’s test (**A**,**C**) and Kruskal-Wallis post-hoc Dunn’s test (**B**).

According to the obtained data, the addition of individual PRR agonists (PLGA-HA-MPLA or PLGA-HA-MDP) to the vaccine formulation resulted in a significantly higher production of total HA-specific IgG antibodies compared to PLGA-HA NPs without any PRR agonists (reciprocal geometric mean titer (GMT) of 800, 174, and 13, respectively) (Figure 8A). However, the most prominent IgG response was observed in mice immunized with PLGA particles containing both PRR agonists (5571). Further IgG subclass response analysis revealed the maximal production of all three measured IgG subclasses in the PLGA-HA-MPLA + MDP-vaccinated group. Adjuvantation with a complex adjuvant results in a significant elevation of IgG1 (7351), IgG2a (9700), and IgG2b (6400) titers compared to vaccine formulations adjuvanted with individual MPLA (4850, 606, and 800, respectively) or MDP (174, 132, and 200, respectively) (Figure 8B). It is worth noting that the difference in IgG1 titers between PLGA-HA-MPLA + MDP (7351) and PLGA-HA-MDP (174) was significant, but not between PLGA-HA-MPLA + MDP (7351) and PLGA-HA-MPLA (4850). As a result, only PLGA NPs containing both PRR agonists showed a ratio of IgG2a/IgG1 antibodies >1 (1.4) compared to control NPs containing individual MDP or MPLA molecules (0.1 and 0.3, respectively) (Figure 8B). Consistent with IgG titers, PLGA-HA-MPLA + MDP NPs induced significantly higher HI responses (reciprocal GMT: 211) compared to the PLGA-HA (106), PLGA-HA-MDP (106), and PLGA-HA-MPLA (121) NPs (Figure 8C).

Thus, the PLGA NPs containing both TLR4 and NOD2 agonists enhances the magnitude of all studied parameters of anti-influenza humoral response compared to control NPs containing individual PRR agonists.

### 3.9. PLGA NPs Containing a Combination of TLR4 and NOD2 Agonists Lead to a Stronger Protection in Mice than NPs Containing Individual PRR Agonists

**Figure 9:**
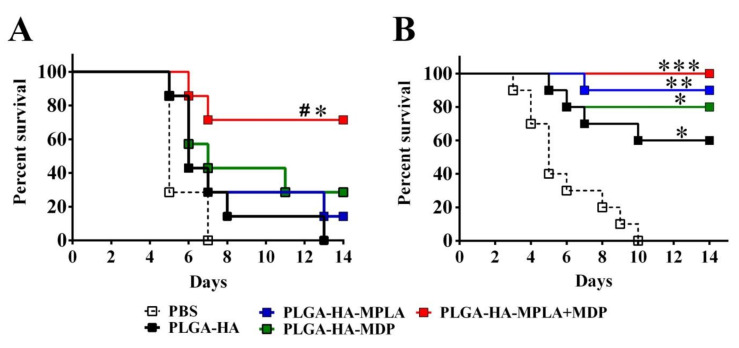
PLGA NPs containing both MPLA and MDP led to a stronger protection in mice than NPs containing individual PRR agonists. Kaplan–Meier survival curves of mice i.m. immunized three times with (**A**) 1 dose (1 mg/mouse, *n* = 8 per group) or (**B**) 2 doses (2 mg/mouse, *n* = 10 per group) of PLGA NPs: PLGA-HA, PLGA-HA-MPLA, PLGA-HA-MDP, and PLGA-HA-MDP-MPLA. Non-immunized control mice received PBS. Two weeks after last immunization, mice were infected using a lethal dose (50LD50) of the A/California/07/2009 (H1N1) strain virus. Mortality was monitored daily for 14 days post-challenge. ^#^ indicates a significant difference (*p* < 0.05) between PLGA-HA-MPLA+MDP and PLGA-HA vaccinated animals. Significant differences between vaccinated with PLGA NPs and non-vaccinated animals are indicated by asterisks: * for *p* < 0.05 and ** for *p* < 0.01 and *** for *p* < 0.001 (log-rank test).

Finally, we studied whether higher immunogenicity of vaccine formulation containing both PRR agonists translates to better protection rates against lethal influenza infection. Three weeks after three immunizations with one or two doses of studied PLGA NPs, mice were challenged with a lethal dose (50LD50) of A/California/07/2009 (H1N1). The chosen dose of influenza virus caused death in 100% of control mice, PBS-treated mice, and mice immunized with one dose of PLGA NPs without adjuvantation (PLGA-HA) (Figure 9A). Immunization with one dose of PLGA NPs containing individual TLR4 and NOD2 agonists did not result in a significant increase in the survival rate (14% and 28%, respectively), whereas immunization with PLGA NPs containing a combination of TLR4 and NOD2 agonists significantly increased the survival rate (72%) compared to control PBS- and PLGA-HA-treated mice. It is important to note that a similar protection rate using sole hemagglutinin could only be achieved at a dose that was 10-fold higher under the same experimental conditions.

Experiments with two-dose immunizations with PLGA NPs containing individual TLR4 and NOD2 agonists showed 90% and 80% protection, respectively, compared to unvaccinated mice (Figure 9B), whereas a two-fold dose increase resulted in 100% animal protection in the group vaccinated with PLGA-HA-MPLA + MDP.

These data indicate the importance of complex adjuvant encapsulation into PLGA NPs to achieve more potent protection compared to adjuvants based on individual PRR agonists.

## 4. Discussion

In spite of numerous approaches aimed at improvement (e.g., through the use of immunostimulatory molecules, modifications of physicochemical characteristics of vaccine particles, adjustment of vaccination regimens, etc.), the effectiveness of most subunit vaccines (e.g., against tuberculosis or influenza) is still behind those types commonly regarded as the “gold standard”: inactivated or live attenuated [29,30]. The discovery of collaboration between different types of PRRs presents an opportunity to improve the adjuvantation strategy of subunit vaccines by using complex immunoadjuvants based on certain combinations of PAMPs. However, there are still several questions that need to be answered to evaluate the practical perspective of complex PAMP-based adjuvants, including (i) whether synergistic effects after the combined stimulation of PRR receptors during immunization could be translated to enhanced vaccine protective efficacy in lethal infection models; (ii) whether the combined stimulation of PRRs during immunization could increase the immunogenicity of subunit vaccines up to levels comparable to those of ‘full-bodied’ attenuated or inactivated vaccine types; (iii) given the latter is possible, what is the minimal number of PAMPs to be included in subunit vaccine composition that will be sufficient to achieve immunogenicity levels comparable to those of ‘classical’ vaccine types. This work aimed to address these issues.

Antigen engulfment by antigen-presenting cells is a first principal step for the development of immune response to vaccination on a cellular level. It has been previously shown that the stimulation of either TLR4 or NOD2 receptors promotes the phagocytosis process [24,25]. In the present study, we determined that the inclusion of complex immunoadjuvants stimulating both TLR4 and NOD2 receptors into PLGA NPs could further intensify the uptake of vaccine particles compared to individual PRR agonists. It is interesting to note that NF-κB activity by itself could also affect phagocytosis rates independently of PRRs. In particular, IKK1 mutant macrophages enhanced NF-κB activation as characterized by increased phagocytotic ingestion of *E. coli* K-12, whereas in another study, the inhibition of NF-κB significantly blocked the phagocytosis of fluorescently labeled *S. aureus* [31,32]. This raises the question of whether cooperative PRR recognition or subsequent enhanced NF-κB activity is the trigger of the accelerated phagocytosis of PAMP-adjuvanted PLGA NPs. It would also be intriguing to determine how the stimulation of cytoplasmic NOD2, complementing TLR4 signaling, increases the phagocytosis rates of vaccine particles within a few minutes after the addition PLGA NPs to the cells.

Compared with biologic particulates, such as bacterial or even virus pathogens, subunit vaccine particles with a less diverse repertoire of PAMPs are expected to induce lower levels of APC phagocytic activity. Thus, it would be promising to estimate whether the adjuvant potential of a chosen pair of PRR agonists could increase the uptake of vaccine particles and reach the values obtained with biological particulates. As the H1N1 strain of influenza virus is broadly used as a component of several multivalent live attenuated vaccines (Fluenz Tetra, FluMist, Ultravac, etc.), the mouse-adapted A/California/07/2009 virus was taken as an example of the whole-pathogen vaccine. The immunogenicity of the subunit and attenuated vaccines cannot generally be compared directly both in vivo and in vitro, due to the infection process caused by pathogen. However, considering the virus as the particulate matter, in our opinion, phagocytosis studies present at least one of the most reliable conditions to compare the immunogenicity of two vaccine types disregarding the self-replicating ability of the pathogen due to short exposure time. Similarity in size and concentrations as measured by the NTA of PLGA NPs and the influenza virus made it possible to compare the immune effects normalized to a single particle. Serendipitously, adjuvantation using both PRR agonists results in comparable phagocytosis rates of influenza virus particles and PLGA NPs. By contrast, elevated levels of cell response measured by the NF-κB/AP-1-dependent reporter or cytokine expression were found only after the addition of PLGA NPs containing either MPLA alone or in combination with MDP, but not in virus-treated cells. These results allow us to conclude that the expansion of PRR agonists included in the vaccine composition could be a useful tool for promoting the phagocytosis of vaccine particles along with other approaches (e.g., particle size and charge). We also speculate that the adjuvantation strategy of subunit vaccine particles using two types of PRR agonists could be the minimum that is sufficient for imitating a full-bodied viral pathogen structure and, thus, resulting in comparable immunogenicity to inactivated virus particles. However, deeper studies are needed to broaden the relevance of this approach.

In the context of vaccinated organisms, it is considered that the inflammatory reactions observed as first immune events at the site of vaccine injection play an important role in further immune cell recruitment and the development of robust adaptive immune reactions. Since NF-κB is a key proinflammatory transcription factor that is also activated upon the recognition of TLR4 and NOD2 receptors, we used NF-κB-Luc transgenic mice as a readout system to measure the degree of inflammation measured by the NF-κB-dependent expression of luciferase following the injection of particulate vaccine formulations. Consistent with previously published data using an alum-based AS04 adjuvant containing MPLA, the most robust NF-κB activation after the administration of PAMP-containing vaccine formulations was observed locally (at the site of injection and in regional draining lymphatic nodes), which could be attributed to the depot effect of alum and PLGA carriers [33]. However, it is worth noting that the injection of only NPs containing a complex immunoadjuvant results in a significant increase in NF-κB activity of organs distal to the site of injection (spleen and liver), which was not observed after PLGA-based formulations containing individual PRR agonists. It is known that vaccine particles smaller than 200 nm after parental administration could be delivered by a broader repertoire of cell types (additional involvement of lymph node-resident DCs and macrophages complementary to DCs present at the injection site) compared to larger particles [34]. However, we assume that at early time points after vaccine injection, where only small amounts of similarly sized nanoscale PLGA particles are dispersed throughout the body only the synergistic effects after combined stimulation of TLR4 and NOD2 receptors could increase a significant inflammatory response in distant organs. The inflammatory response as measured by NF-κB activity levels was consistent with a broad range of induced CC and CXC chemokines, attracting a diverse repertoire of immune cells including DCs, monocytes, macrophages, and neutrophils [35]. G-CSF produced at both proximal and distal sites could serve also as a homing factor for mononuclear cells from mouse bone marrow [36].

In order to evaluate whether these inflammatory reactions are an attribute of enhanced vaccine efficacy, we evaluated several parameters of both humoral and cellular immunity after immunization with PLGA NPs known to be important in the further protection against influenza virus infection. Immunization with vaccine particles containing complex immunoadjuvant results in maximal titers of HA-specific IgG antibodies (GMT—1:5571) as well as all four IgG subtypes in mouse serum samples. It is worth noting that an IgG2a/IgG1 ratio >1 was observed only in the PLGA-HA-MPLA+MDP immunized group. A previously published study demonstrated that mean IgG titers more than 1:1000, with a shift towards IgG2a, were associated with protection from a lethal dose of A/Netherlands/602/2009 (H1N1) influenza virus [37]. Along with that, this group of mice was characterized by maximal HI titers (GMT—1:211). According to a previous publication using the same virus strain, HI titers of >1:40 were considered as markers of protection [38]. The effect of a complex immunoadjuvant on cellular immunity was clarified by a significant increase of splenic CD4 T-cells that were proliferating and/or activated (measured by IFN-γ production) in response to hemagglutinin restimulation in vitro. Importantly, CD4 T-cell proliferation correlates with neutralizing antibody responses that are both shown to be markers of vaccine protection efficacy [39].

Finally, we have shown that PLGA NPs containing both PRR agonists result in significantly higher survival rates using different vaccination doses in comparison to NPs with individual PAMPs. Ultimately, this work shows that complex PAMP-based adjuvants are a more effective alternative to those based on individual PAMP molecules and present the possibility of boosting vaccine protection efficacy up to 10-fold (measured in antigen dose) over non-adjuvanted formulation. The obtained data also support the idea that next-generation complex immunoadjuvants may help subunit vaccines to enter the competition of immunogenicity with ‘classical’ types along with maintaining high safety characteristics. Along with that, it should be noted that the enhanced immune response induced by combined PRR stimulation during immunization could not solely guarantee enhanced vaccine protection [40]. These data do not deflect from the importance of other vaccine components (primarily the vaccine antigen) nor their characteristics in vaccine design studies. The choice of PRRs and corresponding agonists should also be taken into consideration, due to the ability of distinct PRRs to initiate immune reactions with different specificity [41]. Thus, further studies should aim to elucidate nuances in the reactions of adaptive immunity prompted by different sets of PRR agonists and their utility in vaccines against specific infectious diseases.

## Figures and Tables

**Table 1:** Physico-chemical parameters of vaccine formulations (*n* = 3; Mean ± SD).

Vaccine Group	PLGA Weight per Dose, mg	Diameter, nm		Polydispersity Index, PDI	Average Number of Particles per Dose ± SD, 10^12^	Zeta Potential, mV ± SD	PVA Content (*w/w* %)	Total Hemagglu-Tinin (ng per Dose)	Immuno-Stimulatory Molecules (μg per Dose)	
		DLS	NTA						MPLA	MDP
PLGA-HA	1	143.6 ± 36.1	160.6 ± 56.4	0.014	0.90 ± 0.02	−13.2 ± 5.1	7.0	10.8	-	-
PLGA-HA-MPLA	1	135.3 ± 40.5	158.8 ± 54.5	0.054	0.87 ± 0.06	−11.3 ± 4.2	5.7	9.8	1.1	-
PLGA-HA-MDP	1	145.3 ± 38.9	160.5 ± 47.3	0.042	1.28 ± 0.05	−9.8 ± 5.5	5.7	10.3	-	20.5
PLGA-HA-MPLA + MDP	1	128.7 ± 39.2	156.9 ± 42.5	0.075	1.36 ± 0.05	−9.9 ± 4.3	6.2	9.5	0.9	19.8

## Supplementary Materials

The following are available online at https://www.mdpi.com/2076-393X/8/3/519/s1. Figure S1: Linear part of calibrations curves of the SEAP reporter gene expression in HEK-Blue-hTLR4 (A) and HEK-Blue-hNOD2 (B) cells in response to standard concentrations of MPLA (1.25, 0.625, 0.3125 µg/mL) and MDP (6.25, 3.125 and 1.5625 µg/mL), correspondingly (gray diamonds). Experimental samples of PLGA NPs were added at 1 mg/mL and 100 µg/mL concentrations to the HEK-Blue-hTLR4 cells and HEK-Blue-hNOD2, correspondingly. 18 h later SEAP expression was detected in culture medium samples by measuring optical density at 405nm. Data represent the mean values from triplicates. Linear regression equations (illustrated for each curve with coefficient of determination-R2) were used to calculate PRR content in PLGA NPs. Figure S2: Evaluation of protective efficacy of different doses HA in a BALB/c mouse challenge model. Mice (*n* = 10/group) were i.m. immunized three times with different doses of HA. Controls include non-immunized (PBS treated) mice. Two weeks after the last immunization, mice were infected by a lethal dose (50LD50) of the A/California/07/2009 (H1N1) strain virus. Mortality was monitored daily for 14 days post-challenge. Survival was calculated by Kaplan–Meier analysis. Significant differences between mice vaccinated with HA and unvaccinated (PBS-treated) mice are indicated by hashes: ^#^ for *p* < 0.05 or ^##^ for *p* < 0.01 (log-rank test). Figure S3: Combined stimulation of TLR4 and NOD2 receptors leads to synergistic NF-κB/AP-1-dependent SEAP reporter gene expression in RAW-Blue cells. NF-κB/AP-1 activity (NF-κB/AP-1-dependent SEAP reporter gene expression) in RAW-Blue cells (Invivogen, San Diego, CA, USA) treated for 18 h with the indicated doses (μg/mL) of MDP and MPLA (alone or in combination). Mathematical modeling of synergy was performed basing on previously published method [42] (A) Bars represent a mean fold increase ± SD (error bars) in SEAP activity relative to intact (untreated) cells (values are mean from three independent experiments, each performed in duplicate). Asterisks indicate significant differences (*p* < 0.05) in SEAP activity between treatment with a combination of PRR agonists and the sum of the activity levels seen in cells treated with MPLA and MDP alone. (B) Graphical presentation (matrix and contour plot) of cellular response calculated basing on Zero interaction potency (ZIP) reference model using SynergyFinder [43]. Matrix (upper figure) depicts observed cell responses after individual and combined TLR4 and NOD2 stimulation. Digits in squares and color intensity represent SEAP activity calculated as % of maximal response observed after addition of maximal doses of TLR4 (10 µg/mL) and NOD2 (100µg/mL) agonists. Contour plot (lower figure) depicts calculated ZIP synergy score with positive and negative values denoting synergy and antagonism, respectively. For estimation of outlier measurements cNMF algorithm [44] implemented in SynergyFinder was utilized. Yellow square depicts synergy score achieved using combination of TLR4 and NOD2 agonists at doses 1µg/mL and 20µg/mL, respectively, chosen for encapsulation into single dose of PLGA NPs. Figure S4: Negative-stained TEM image of spherical monodisperse hemagglutinin loaded poly (lactic-co-glycolic acid) nanoparticles adjuvanted with MDP and MPLA (PLGA-HA-MDP-MPLA). Scale bar: 200 nm. The ultrastructure of PLGA NPs was studied using a negative staining method with a transmission electron microscope. A drop of suspension with PLGA NPs was placed on a formvar copper grid (SPI Supplies, USA) for 40 s. Nonadherent particles were removed by filter paper. A copper grid with PLGA NPs was stained with 1% uranyl acetate for 30 s, and the excess of stain was then removed by filter paper. Grids were dried at room temperature for 10 min and analyzed using transmission electron microscope CM-12 (Phillips, Eindhoven, The Netherlands) at an accelerating voltage of 80 kV. Figure S5: Characterization of PLGA and A/California/07/2009 (H1N1) virus particles stained with pHRodo. (A) The representative curve of the size distribution of PLGA-HA-MPLA + MDP NPs and influenza viral particles. The quantity of virus and PLGA particles as well as the size distributions were estimated by nanoparticle tracking analysis (NTA) using a NanoSight NS300 system. Prior to the staining of influenza virus with pHRodo dye, the concentration of influenza virus was normalized up to 10^12^ vp/mL to the previously obtained PLGA NPs containing HA stained with pHRodo. (B) Relative fluorescence units (RFU) of virus samples stained with pHRodo at the indicated concentrations. Each bar represents the mean fluorescence of the corresponding sample ± SD. Staining index (mean fluorescence/particle number) of each sample is shown above the bar. Staining of the influenza virus with 12.5 μM pHRodo yielded the staining index closest to PLGA-HA-MPLA+MDP (underlined). This influenza virus was used for phagocytosis studies. (C) Characteristics of the stained PLGA and virus particles used for phagocytosis studies. Size shown as mean ± SD. Figure S6. Cytokine levels in cell-free culture supernatants from the BMDCs treated with PLGA NPs containing combination of PRR agonists in comparison to mixture of PLGA NPs containing individual PRR agonists taken in equal proportion. Values of control group (mean concentrations ± SD are indicated above the bars) were taken equal to 1 (100%). Data represent the mean fold increase over intact cells ± SD. Significant differences between PLGA NPs and intact cells are indicated by hashes: ^#^ for *p* < 0.05, ^##^ for *p* < 0.01, or ^###^ for p < 0.001, ^####^ for *p* < 0.0001 (one-way ANOVA with Dunnett’s post hoc test). Significant differences between PLGA treated groups are indicated by asterisks and brackets: * for *p* < 0.05, ** for *p* < 0.01, or *** for *p* < 0.001, **** for *p* < 0.0001 (one-way ANOVA with Dunnett’s post hoc test). Table S1. Complete blood count (CBC) parameters of 8-week-old BALB/c mice 2 days after i.m. injection of prepared vaccine PLGA NPs (1mg and 2mg per dose) and pharmaceutical grade Aluminium hydroxide Vac20 type (SPI Pharma, Wilmington, DE, USA). Mice in this experiment have not taken part in other experiments. Aluminium hydroxide was taken as a reference adjuvant composition in average dose (650 µg) used for human [Biotecnología Aplicada 2013;30:250–256]. Non-immunized control mice received 0.9% sodium chloride. Blood from tail vein was collected in EDTA containing tubes. CBC analysis was performed using Mythic 18 Vet Haematology Analyser (Woodley Equipment, Bolton, UK). Leukocyte populations were counted in blood smears after May-Grünwald staining. Data are given as mean ± SD. Number of mice in each group is shown in corresponding column. Bold and underlined numbers in experimental groups represent statistically significant (* *p* < 0.05, nonparametric Kruskal–Wallis test) increase over the control group. Table S2. Complete blood count (CBC) parameters of 8-week-old BALB/c mice 14 days after i.m. injection of prepared vaccine PLGA NPs (1mg and 2mg per dose) and pharmaceutical grade Aluminium hydroxide Vac20 type (SPI Pharma, Wilmington, DE, USA). Mice in this experiment have not taken part in other experiments. Aluminium hydroxide was taken as a reference adjuvant composition in average dose (650µg) used for human [Biotecnología Aplicada 2013;30:250–256]. Non-immunized control mice received 0.9% sodium chloride. Blood from tail vein was collected in EDTA containing tubes. CBC analysis was performed using Mythic 18 Vet Haematology Analyser (Woodley Equipment, Bolton, UK, UK). Leukocyte populations were counted in blood smears after May-Grünwald staining. Data are given as mean ± SD. Number of mice in each group is shown in corresponding column. Table S3. Routine biochemistry parameters in blood serum of 8-week-old BALB/c mice 14 days after i.m. injection of prepared vaccine PLGA NPs (1mg and 2 mg per dose) and pharmaceutical grade Aluminium hydroxide Vac20 type (SPI Pharma, Wilmington, DE, USA). Mice in this experiment have not taken part in other experiments. Aluminium hydroxide was taken as a reference adjuvant composition in average dose (650 µg) used for human [Biotecnología Aplicada 2013;30:250–256]. Non-immunized control mice received 0.9% sodium chloride. Serum was prepared from blood collected from tail vein. Concentration of main clinically important molecules and ions were measured using corresponding reagent kits (Randox Laboratories, Crumlin, UK) and Sapphire 400 biochemical analyzer (Tokyo Boeki LTD, Kyobashi, Japan). Data are given as mean ± SD. Number of mice in each group is shown in corresponding column. Bold and underlined numbers in experimental groups represent statistically significant (* *p* < 0.05, nonparametric Kruskal-Wallis test) increase over the control group.

## Patents

The authors declare the following patent relating to material pertinent to this article: The use of monophosporyl lipid A (MPLA) and muramyldipeptide (MDP) as a complex vaccine adjuvant (RU 2497541 C1). There are no further patents, products in development or marketed products to declare.
