# Supplementary material for: Adjuvantation of an Influenza Hemagglutinin Antigen with TLR4 and NOD2 Agonists Encapsulated in Poly(D,L-Lactide-Co-Glycolide) Nanoparticles Enhances Immunogenicity and Protection against Lethal Influenza Virus Infection in Mice
Source: Vaccines (Basel). 2020 Sep 10;8(3):519. doi: 10.3390/vaccines8030519 (PMC7564367; doi:10.3390/vaccines8030519)
Supplement: Supplementary file 1 [file vaccines-08-00519-s001.zip › vaccines-918746-xml-sup/Supplementary tables 1,2,3.docx]

**Table S1.** Complete blood count (CBC) parameters of 8 week old BALB/c mice 2 days after i.m. injection of prepared vaccine PLGA NPs (1mg and 2mg per dose) and pharmaceutical grade Aluminium hydroxide Vac20 type (SPI Pharma. USA). Mice in this experiment have not taken part in other experiments. Aluminium hydroxide was taken as a reference adjuvant composition in average dose (650µg) used for human [Biotecnología Aplicada 2013;30:250-256]. Non-immunized control mice received 0.9% sodium chloride. Blood from tail vein was collected in EDTA containing tubes. CBC analysis was performed using Mythic 18 Vet Haematology Analyser (Woodley Equipment, UK). Leukocyte populations were counted in blood smears after May-Grünwald staining. Data are given as mean ± SD. Number of mice in each group is shown in corresponding column. Bold and underlined numbers in experimental groups represent statistically significant (* p < 0.05, nonparametric Kruskal-Wallis test) increase over the control group.

| CBC parameters | 0.9% sodium chloride | | PLGA-HA-MPLA+  MDP, 1mg/dose | | PLGA-HA-MPLA+  MDP, 2mg/dose | | Alum, 600µg/dose | |
| --- | --- | --- | --- | --- | --- | --- | --- | --- |
|  | males (n=5) | females  (n=5) | males (n=5) | females  (n=5) | males (n=5) | females  (n=5) | males (n=5) | females  (n=5) |
| Hemoglobin (Hb), g/L | 148±11 | 152±9 | 142±8 | 152±2 | 147±8 | 145±19 | 149±5 | 156±13 |
| Red blood cells (RBC), x10^12^/L | 9.46±0.76 | 9.60±0.29 | 8.92±0.52 | 9.70±0.14 | 9.32±0.40 | 9.09±1.16 | 9.51±0.27 | 10.02±0.97 |
| Hematocrit (HCT), % | 0.459±0.035 | 0.477±0.027 | 0.438±0.023 | 0.479±0.005 | 0.454±0.022 | 0.450±0.055 | 0.464±0.018 | 0.502±0.050 |
| Mean corpuscular volume (MCV), fl | 48.6±0.8 | 49.7±1.8 | 49.1±1.1 | 49.4±1.2 | 48.7±0.5 | 49.6±0.8 | 48.8±0.7 | 50.1±0.5 |
| Mean corpuscular hemoglobin (MCH), pg | 15.6±0.3 | 15.9±0.6 | 16.0±0.3 | 15.7±0.2 | 15.7±0.3 | 16.0±0.5 | 15.7±0.3 | 15.6±0.3 |
| Mean corpuscular hemoglobin concentration (MCHC), g/l | 322±3 | 319±5 | 325±5 | 317±6 | 323±6 | 322±9 | 322±7 | 311±9 |
| Red cell distribution width expressed as coefficient of variation (RDW-CV), % | 14.5±0.7 | 14.6±0.7 | 14.9±0.9 | 14.5±0.4 | 14.7±0.6 | 15.1±1.4 | 14.4±0.4 | 14.9±0.7 |
| Platelets, x10^9^/L | 733±54 | 599±118 | 722±123 | 693±65 | 728±86 | 650±83 | 727±73 | 625±76 |
| White blood cells, x10^9^/L | 3.3±0.7 | 3.5±1.1 | 2.6±0.7 | 3.0±1.3 | 2.5±0.6 | 2.9±0.6 | 3.2±0.4 | 4.2±1.5 |
| Lymphocytes, x10^9^/L | 2.91±0.71 | 3.15±1.07 | 2.29±0.58 | 2.67±1.22 | 2.10±0.55 | 2.45±0.56 | 2.65±0.36 | 3.48±1.23 |
| Basophils, x10^9^/L | 0.00±0.00 | 0.00±0.00 | 0.00±0.00 | 0.00±0.00 | 0.00±0.00 | 0.00±0.00 | 0.00±0.00 | 0.00±0.00 |
| Eosinophils, x10^9^/L | 0.03±0.03 | 0.02±0.02 | 0.01±0.02 | 0.03±0.01 | 0.03±0.01 | 0.01±0.01 | 0.03±0.01 | 0.04±0.01 |
| Monocytes, x10^9^/L | 0.05±0.02 | 0.06±0.02 | 0.03±0.01 | 0.03±0.01 | 0.03±0.01 | 0.05±0.02 | 0.05±0.02 | 0.04±0.01 |
| Band neutrophils, x10^9^/L | 0.00±0.00 | 0.00±0.00 | 0.01±0.01 | 0.00±0.00 | 0.00±0.00 | 0.00±0.00 | 0.00±0.00 | 0.00±0.00 |
| Segmented neutrophils, x10^9^/L | 0.32±0.06 | 0.29±0.09 | 0.28±0.11 | 0.25±0.06 | 0.35±0.11 | 0.35±0.07 | ***0.45±0.06** | 0.62±0.21 |

**Table S2.** Complete blood count (CBC) parameters of 8 week old BALB/c mice 14 days after i.m. injection of prepared vaccine PLGA NPs (1mg and 2mg per dose) and pharmaceutical grade Aluminium hydroxide Vac20 type (SPI Pharma. USA). Mice in this experiment have not taken part in other experiments. Aluminium hydroxide was taken as a reference adjuvant composition in average dose (650µg) used for human [Biotecnología Aplicada 2013;30:250-256]. Non-immunized control mice received 0.9% sodium chloride. Blood from tail vein was collected in EDTA containing tubes. CBC analysis was performed using Mythic 18 Vet Haematology Analyser (Woodley Equipment, UK). Leukocyte populations were counted in blood smears after May-Grünwald staining. Data are given as mean ± SD. Number of mice in each group is shown in corresponding column.

| CBC parameters | 0.9% sodium chloride | | PLGA-HA-MPLA+  MDP, 1mg/dose | | PLGA-HA-MPLA+  MDP, 2mg/dose | | Alum, 600µg/dose | |
| --- | --- | --- | --- | --- | --- | --- | --- | --- |
|  | males (n=6) | females  (n=6) | males (n=6) | females  (n=5) | males (n=6) | females  (n=6) | males (n=6) | females  (n=6) |
| Hemoglobin (Hb), g/L | 148±3 | 155±9 | 149±10 | 148±6 | 149±5 | 150±9 | 145±6 | 162±7 |
| Red blood cells (RBC), x10^12^/L | 8.91±0.29 | 9.04±0.48 | 9.11±0.70 | 8.99±0.16 | 8.98±0.44 | 9.05±0.63 | 8.98±0.50 | 9.62±0.62 |
| Hematocrit (HCT), % | 0.436±0.013 | 0.453±0.025 | 0.444±0.031 | 0.448±0.012 | 0.440±0.020 | 0.459±0.036 | 0.439±0.025 | 0.489±0.031 |
| Mean corpuscular volume (MCV), fl | 49.0±0.4 | 50.1±0.9 | 48.8±0.5 | 49.9±0.8 | 49.0±0.4 | 50.7±0.6 | 48.9±0.6 | 50.8±0.8 |
| Mean corpuscular hemoglobin (MCH), pg | 16.6±0.5 | 17.2±0.5 | 16.4±0.3 | 16.5±0.6 | 16.6±0.4 | 16.6±0.4 | 16.2±0.6 | 16.8±0.5 |
| Mean corpuscular hemoglobin concentration (MCHC), g/l | 339±10 | 342±7 | 336±7 | 330±13 | 338±6 | 327±10 | 330±10 | 331±10 |
| Red cell distribution width expressed as coefficient of variation (RDW-CV), % | 15.5±0.7 | 16.0±0.6 | 15.5±0.3 | 15.7±0.5 | 15.4±0.4 | 16.3±0.5 | 15.1±0.6 | 15.7±0.9 |
| Platelets, x10^9^/L | 748±98 | 664±56 | 805±85 | 706±55 | 859±55 | 680±61 | 707±154 | 645±107 |
| White blood cells, x10^9^/L | 2.7±1.0 | 4.1±2.1 | 4.2±2.0 | 3.0±0.8 | 3.6±1.5 | 2.9±0.9 | 2.5±1.0 | 3.4±1.7 |
| Lymphocytes, x10^9^/L | 2.32±0.82 | 3.73±1.85 | 3.65±1.80 | 2.73±0.73 | 3.14±1.20 | 2.51±0.73 | 2.25±0.91 | 2.89±1.46 |
| Basophils, x10^9^/L | 0.00±0.00 | 0.0±0.0 | 0.00±0.00 | 0.0±0.0 | 0.00±0.00 | 0.0±0.0 | 0.00±0.00 | 0.0±0.0 |
| Eosinophils, x10^9^/L | 0.03±0.02 | 0.07±0.12 | 0.05±0.03 | 0.03±0.01 | 0.04±0.02 | 0.04±0.02 | 0.02±0.01 | 0.03±0.02 |
| Monocytes, x10^9^/L | 0.04±0.02 | 0.05±0.02 | 0.05±0.03 | 0.04±0.02 | 0.05±0.04 | 0.03±0.01 | 0.03±0.01 | 0.03±0.02 |
| Band neutrophils, x10^9^/L | 0.0±0.0 | 0.00±0.00 | 0.0±0.0 | 0.00±0.00 | 0.0±0.0 | 0.00±0.00 | 0.0±0.0 | 0.00±0.00 |
| Segmented neutrophils, x10^9^/L | 0.32±0.13 | 0.21±0.13 | 0.49±0.25 | 0.24±0.13 | 0.37±0.28 | 0.31±0.17 | 0.19±0.12 | 0.43±0.26 |

**Table S3.** Routine biochemistry parameters in blood serum of 8 week old BALB/c mice 14 days after i.m. injection of prepared vaccine PLGA NPs (1mg and 2mg per dose) and pharmaceutical grade Aluminium hydroxide Vac20 type (SPI Pharma. USA). Mice in this experiment have not taken part in other experiments. Aluminium hydroxide was taken as a reference adjuvant composition in average dose (650µg) used for human [Biotecnología Aplicada 2013;30:250-256]. Non-immunized control mice received 0.9% sodium chloride. Serum was prepared from blood collected from tail vein. Concentration of main clinically important molecules and ions were measured using corresponding reagent kits (Randox Laboratories, United Kingdom) and Sapphire 400 biochemical analyzer (Tokyo Boeki LTD, Japan). Data are given as mean ± SD. Number of mice in each group is shown in corresponding column. Bold and underlined numbers in experimental groups represent statistically significant (* p < 0.05, nonparametric Kruskal-Wallis test) increase over the control group.

| Serum parameters | 0.9% sodium chloride (control group) | | PLGA-HA-MPLA+  MDP, 1mg/dose | | PLGA-HA-MPLA+  MDP, 2mg/dose | | Aluminium hydroxide, 650µg/dose | |
| --- | --- | --- | --- | --- | --- | --- | --- | --- |
|  | males (n=4) | females  (n=6) | males  (n=6) | females  (n=6) | males  (n=6) | females  (n=6) | males  (n=5) | females  (n=6) |
| Urea, mmol/L | 8.9 ± 1.4 | 9.8 ± 2.7 | 10.3 ± 1.5 | 10.1 ± 2.6 | 10.6 ± 2.2 | 10.9 ± 1.8 | 10.4 ± 3.0 | 9.7 ± 1.3 |
| Cholesterol, mmol/L | 2.9 ± 0.3 | 2.3 ± 0.1 | 2.8 ± 0.3 | 2.2 ± 0.2 | 2.8 ± 0.1 | 2.3 ± 0.2 | 2.7 ± 0.2 | 2.5 ± 0.2 |
| Triglyceride, mmol/L | 1.0 ± 0.1 | 1.0 ± 0.5 | 1.2 ± 0.1 | 0.9 ± 0.2 | 1.3 ± 0.6 | 1.0 ± 0.3 | 1.0 ± 0.4 | 0.9 ± 0.2 |
| ALT. U/L | 41.6 ± 6.3 | 36.6 ± 4.7 | 47.9 ± 17.9 | 38.4 ± 9.3 | 47.8 ± 17.1 | 39.1 ± 4.2 | 53.7 ± 30.2 | 48.2 ± 24.2 |
| AST, U/L | 66.3 ± 31.8 | 62.6 ± 9.9 | 57.7 ± 11.5 | 72.6 ± 13.0 | 64.1 ± 27.3 | 68.5 ± 4.8 | 58.5 ± 16.9 | 83.3 ± 33.1 |
| Bilirubin, mmol/L | 6.4 ± 1.2 | 3.6 ± 0.9 | 6.2 ± 0.9 | 3.5 ± 2.0 | 6.0 ± 1.2 | 4.0 ± 0.5 | 5.7 ± 0.8 | 2.8 ± 1.8 |
| Creatinine, µmol/L | 41.8 ± 3.3 | 46.3 ± 6.0 | 38.5 ± 6.5 | 42.3 ± 4.8 | 43.5 ± 4.2 | 45.0 ± 5.3 | 38.4 ± 4.7 | 46.2 ± 5.0 |
| ALP, U/L | 130.8 ± 7.6 | 135.5 ± 12.0 | 124.0 ± 8.5 | 135.0 ± 9.4 | 137.5 ± 9.4 | 143.7 ± 12.0 | 126.4 ± 7.8 | 138.7 ± 11.7 |
| Albumin, g/l | 26.4 ± 1.0 | 28.9 ± 1.4 | 26.8 ± 2.0 | 28.8 ± 2.0 | 26.5 ± 0.5 | 29.0 ± 1.5 | 27.3 ± 1.6 | 31.0 ± 2.0 |
| Calcium, mmol/L | 2.0 ± 0.1 | 2.1 ± 0.1 | 2.0 ± 0.1 | 2.1 ± 0.0 | 2.0 ± 0.1 | 2.1 ± 0.1 | 2.1 ± 0.1 | 2.1 ± 0.1 |
| Phosphate, mmol/L | 2.4 ± 0.5 | 2.8 ± 0.5 | 2.9 ± 0.4 | 3.0 ± 0.2 | 2.7 ± 0.5 | ***3.5 ± 0.2** | 2.8 ± 0.4 | 3.0 ± 0.5 |
| Total protein, g/l | 41.9 ± 0.7 | 42.7 ± 1.4 | 41.9 ± 1.9 | 42.0 ± 2.6 | 42.8 ± 1.0 | 43.1 ± 0.9 | 42.3 ± 1.2 | 44.3 ± 3.5 |
| Chloride, mmol/L | 108.5 ± 6.5 | 118.2 ± 7.2 | 111.7 ± 9.4 | 119.8 ± 8.0 | 105.3 ± 5.6 | 118.8 ± 10.7 | 115.4 ± 9.2 | 125.5 ± 4.5 |
| Globulins, g/l | 15.5 ± 1.2 | 13.8 ± 1.1 | 15.2 ± 0.5 | 13.2 ± 1.2 | 16.3 ± 1.2 | 14.1 ± 0.7 | 15.0 ± 1.0 | 13.3 ± 1.7 |
| Albumin:globulin ratio | 1.7 ± 0.2 | 2.1 ± 0.2 | 1.8 ± 0.2 | 2.2 ± 0.2 | 1.6 ± 0.2 | 2.1 ± 0.2 | 1.8 ± 0.2 | 2.3 ± 0.2 |
