# Supplementary figures and images for: Adjuvantation of an Influenza Hemagglutinin Antigen with TLR4 and NOD2 Agonists Encapsulated in Poly(D,L-Lactide-Co-Glycolide) Nanoparticles Enhances Immunogenicity and Protection against Lethal Influenza Virus Infection in Mice
Source: Vaccines (Basel). 2020 Sep 10;8(3):519. doi: 10.3390/vaccines8030519 (PMC7564367; doi:10.3390/vaccines8030519)

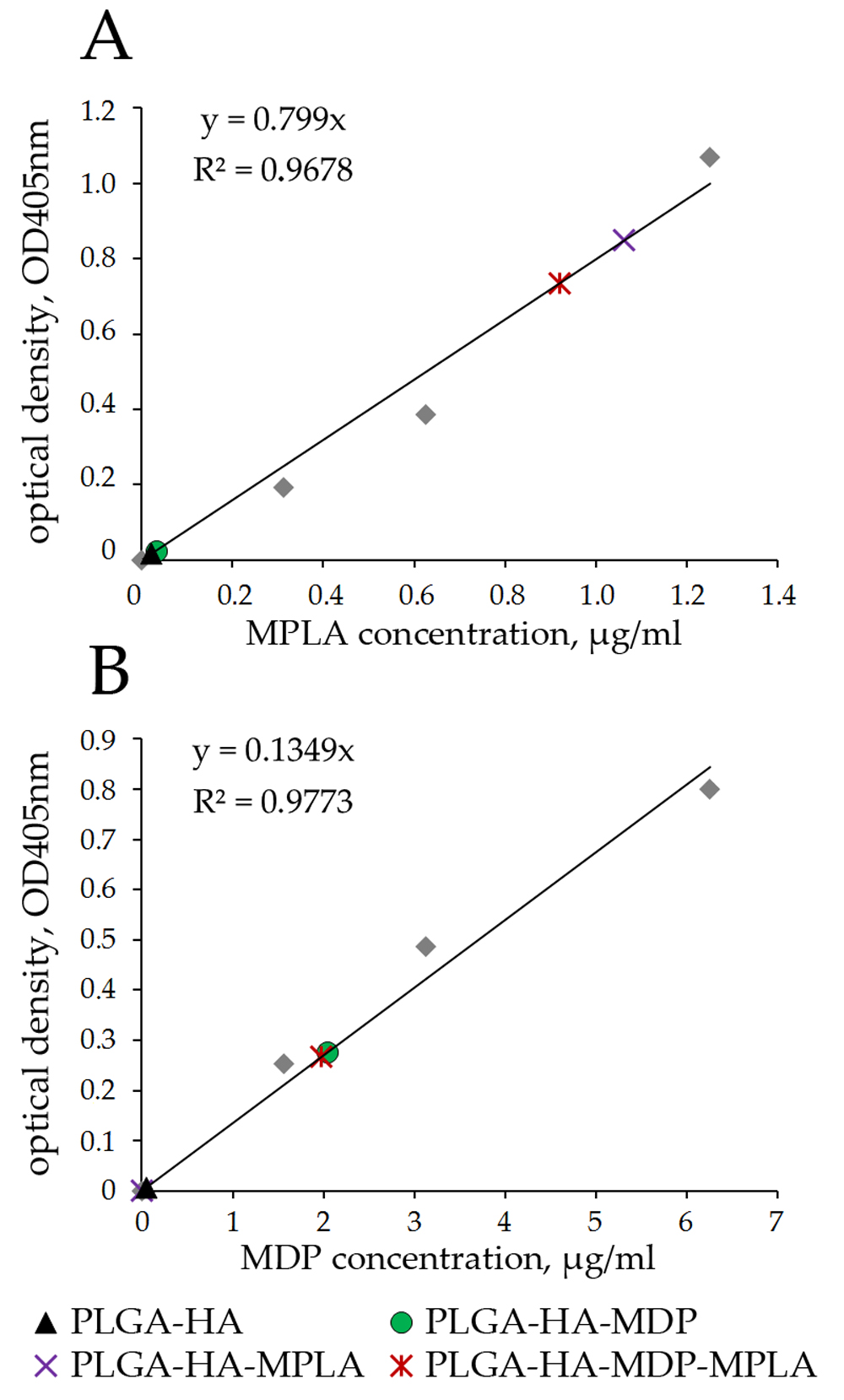

Supplement: Supplementary file 1 [file vaccines-08-00519-s001.zip › Figure S1.jpg]

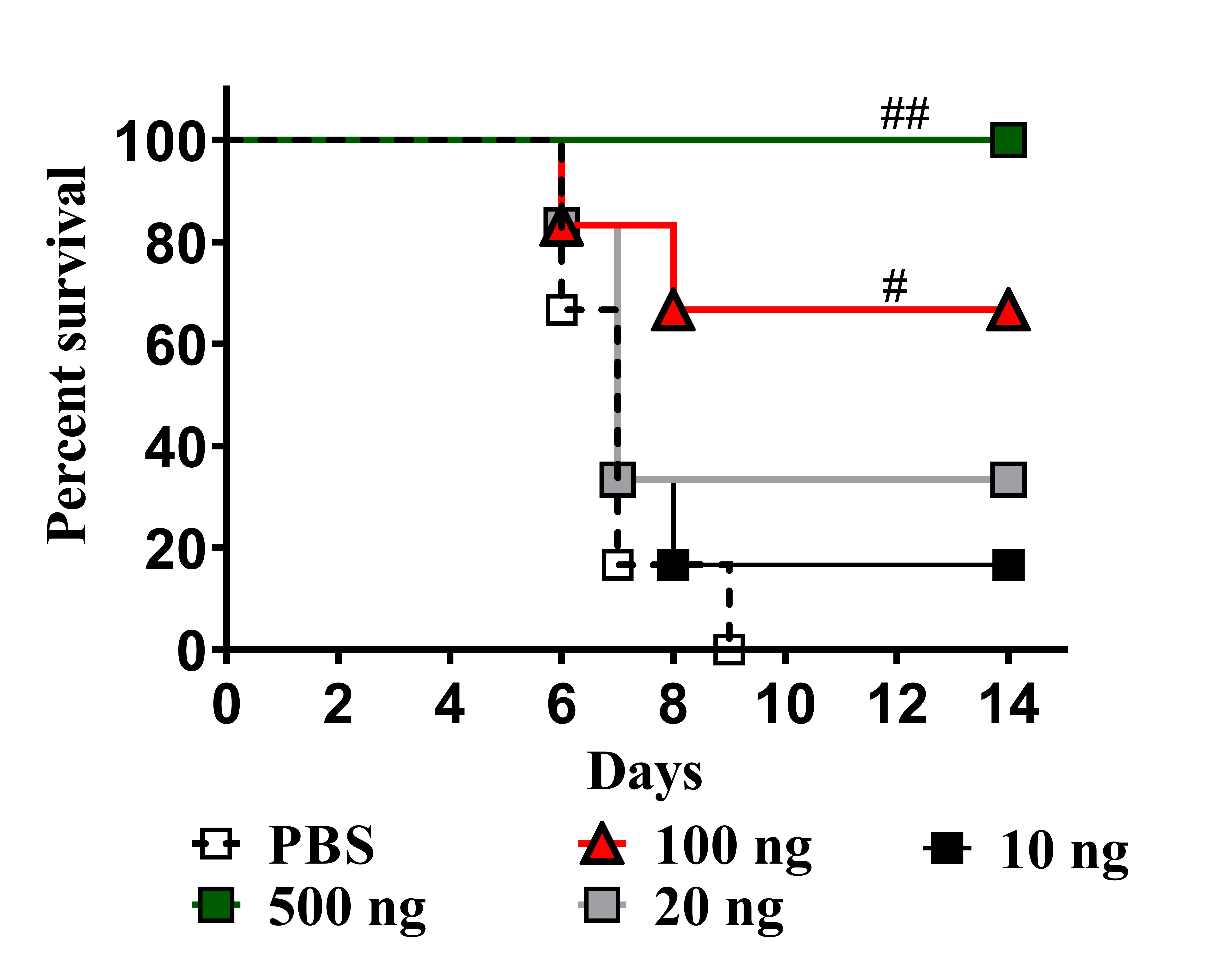

Supplement: Supplementary file 1 [file vaccines-08-00519-s001.zip › Figure S2.jpg]

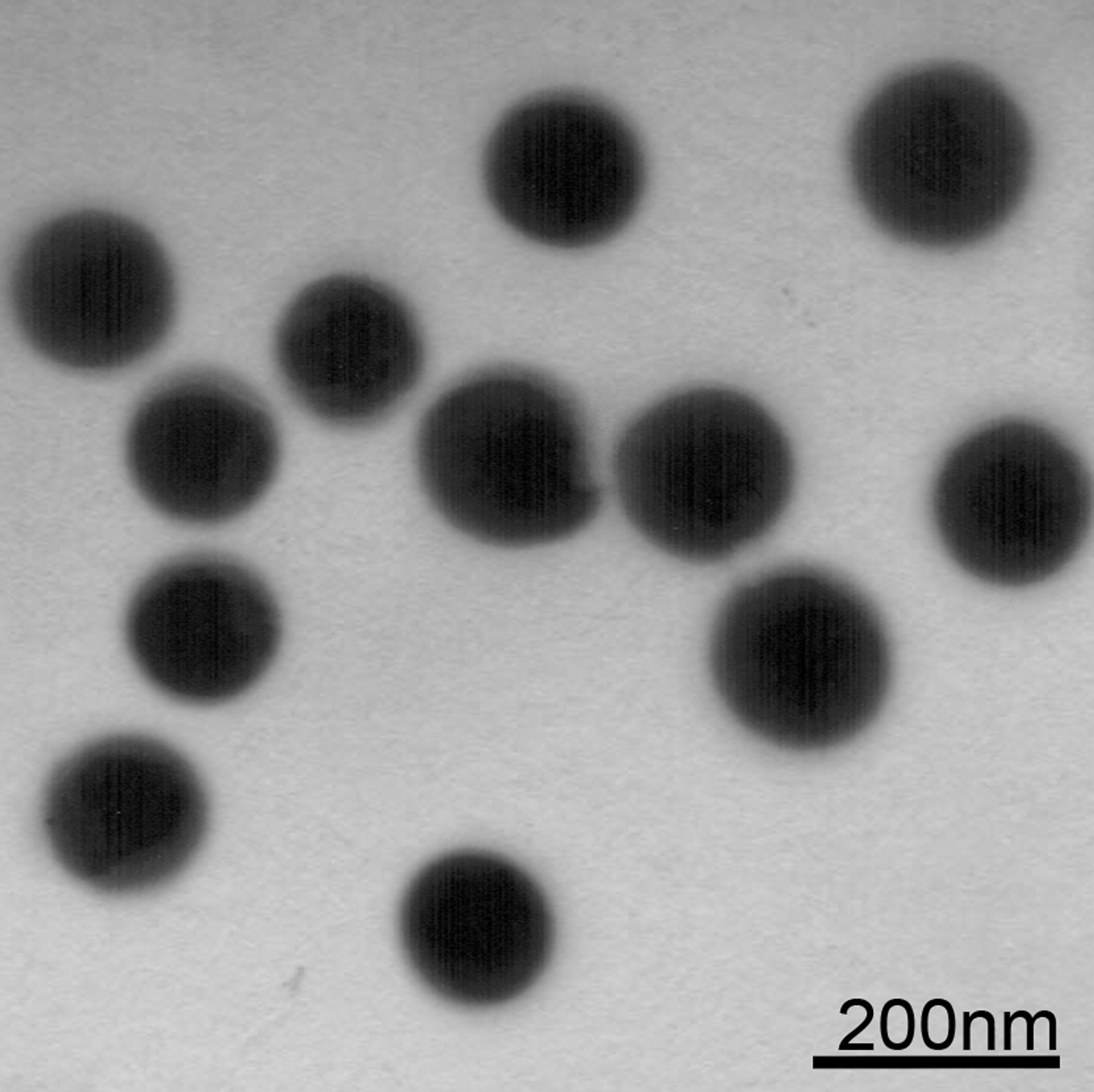

Supplement: Supplementary file 1 [file vaccines-08-00519-s001.zip › Figure S4.jpg]

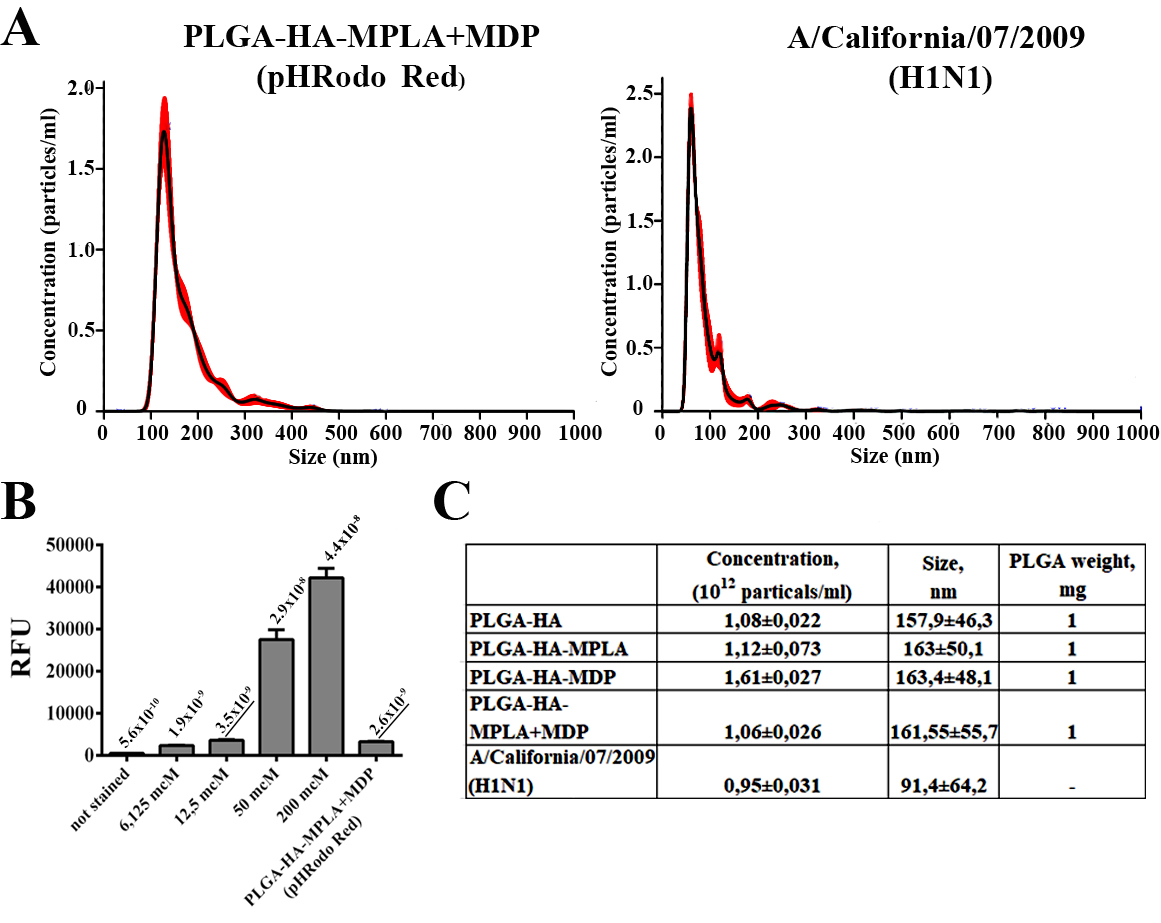

Supplement: Supplementary file 1 [file vaccines-08-00519-s001.zip › Figure S5.jpg]

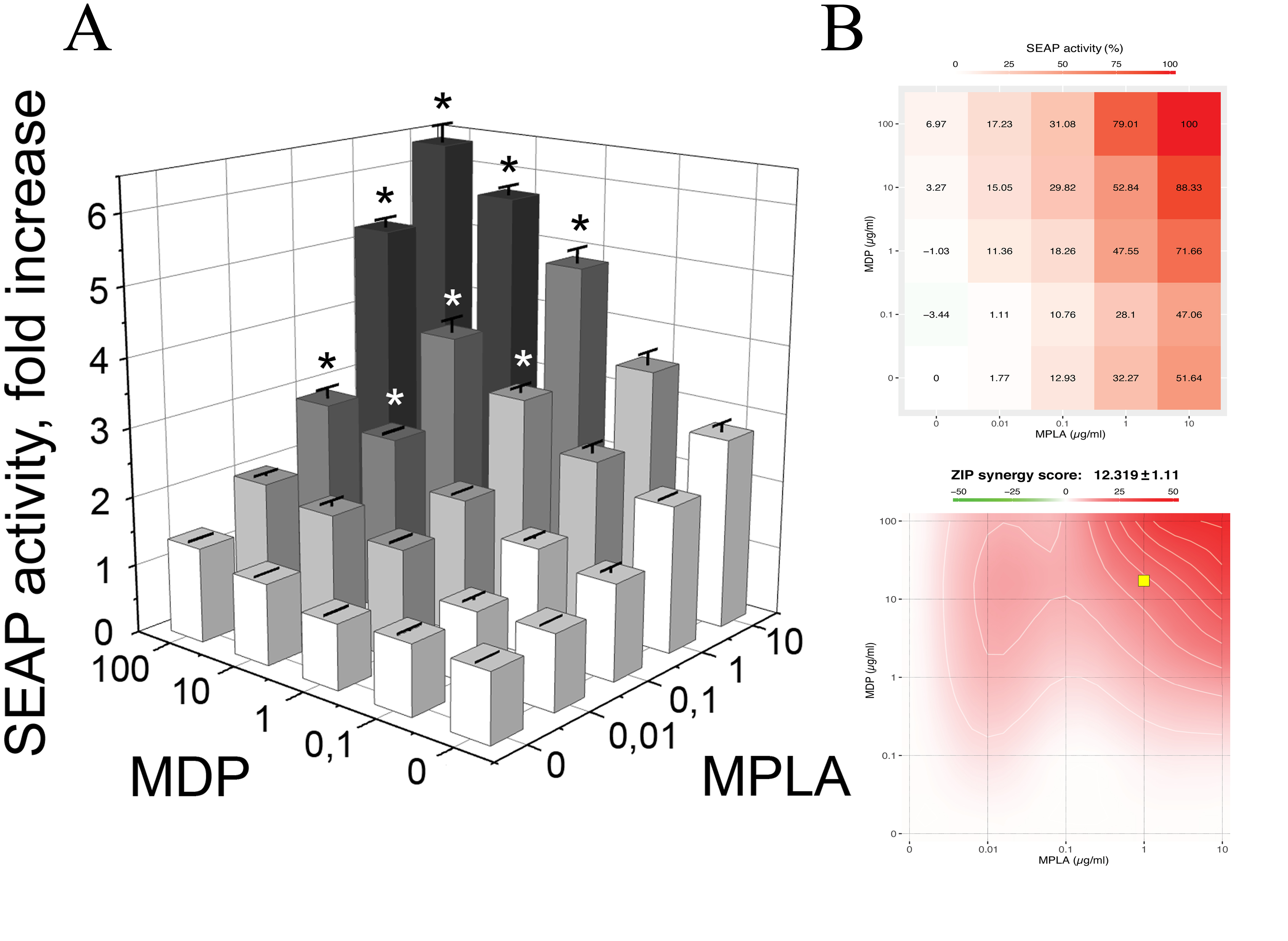

Supplement: Supplementary file 1 [file vaccines-08-00519-s001.zip › Fugure S3.jpg]
